# X-ray-induced piezoresponse during X-ray photon correlation spectroscopy of PbMg_1/3_Nb_2/3_O_3_

**DOI:** 10.1107/S1600577523009116

**Published:** 2024-01-01

**Authors:** Dina Sheyfer, Hao Zheng, Matthew Krogstad, Carol Thompson, Hoydoo You, Jeffrey A. Eastman, Yuzi Liu, Bi-Xia Wang, Zuo-Guang Ye, Stephan Rosenkranz, Daniel Phelan, Eric M. Dufresne, G. Brian Stephenson, Yue Cao

**Affiliations:** a Materials Science Division, Argonne National Laboratory, Lemont, IL 60439, USA; b X-ray Science Division, Argonne National Laboratory, Lemont, IL 60439, USA; c Department of Physics, Northern Illinois University, DeKalb, IL 60115, USA; d Center for Nanoscale Materials, Argonne National Laboratory, Lemont, IL 60439, USA; e Department of Chemistry and 4D Labs, Simon Fraser University, Burnaby, BC, Canada V5A1S6; University of Malaga, Spain

**Keywords:** X-ray photon correlation spectroscopy, relaxor, surface charging, X-ray-induced piezoresponse

## Abstract

X-ray photon correlation spectroscopy (XPCS) holds strong promise for observing atomic-scale dynamics in materials, both at equilibrium and during non-equilibrium transitions. Here an *in situ* XPCS study of the relaxor ferroelectric PbMg_1/3_Nb_2/3_O_3_ (PMN) is reported. A weak applied AC electric field generates strong response in the speckle of the diffuse scattering from the polar nanodomains, which is captured using the two-time correlation function. Correlated motions of the Bragg peak are also observed, which indicate dynamic tilting of the illuminated volume. This tilting quantitatively accounts for the observed two-time speckle correlations. The magnitude of the tilting would not be expected solely from the modest applied field, since PMN is an electrostrictive material with no linear strain response to the field. A model is developed based on non-uniform static charging of the illuminated surface spot by the incident micrometre-scale X-ray beam and the electrostrictive material response to the combination of static and dynamic fields. The model qualitatively explains the direction and magnitude of the observed tilting, and predicts that X-ray-induced piezoresponse could be an important factor in correctly interpreting results from XPCS and nanodiffraction studies of other insulating materials under applied AC field or varying X-ray illumination.

## Abstract 2

X-ray illumination induces surface charging and gives rise to substantially enhanced piezoresponse in a relaxor ferroelectric captured by X-ray photon correlation spectroscopy.

## 1. Introduction

The advent of coherent X-rays at synchrotrons and free-electron laser facilities has provided novel tools for investigating the evolution of spatial heterogeneities in materials. Of particular interest is X-ray photon correlation spectroscopy (XPCS) (Shpyrko, 2014 ▸; Sandy *et al.*, 2018 ▸), in which the dynamics of a speckle pattern are analyzed to characterize dynamic processes in the material. In the wide-angle scattering geometry, XPCS is sensitive to atomic-scale dynamics. It promises to be a powerful method to capture the evolution of spatial heterogeneities in a wide range of quantum and functional materials (Shpyrko *et al.*, 2007 ▸; Su *et al.*, 2012 ▸; Chen *et al.*, 2019 ▸; Robinson *et al.*, 2020 ▸; Shen *et al.*, 2021 ▸), both at equilibrium and in response to stimuli.

One of the most iconic examples of a heterogeneous material is the relaxor class of ferroelectrics. These materials exhibit large, temperature- and frequency-dependent dielectric constants (Cowley *et al.*, 2011 ▸; Bokov & Ye, 2006 ▸). In the canonical relaxor PbMg_1/3_Nb_2/3_O_3_ (PMN), the dielectric constant is maximum near room temperature (Westphal *et al.*, 1992 ▸; Bokov & Ye, 2006 ▸; Ye & Schmid, 1993 ▸). While the average atomic structure of PMN remains cubic with zero net polarization, structural heterogeneities arise due to broken local symmetry arising from chemical and polar disorder. Prevailing hypotheses of the relaxor behavior involve the existence of spatially inhomogeneous polarization, including the formation of polar nanoregions or low-energy domain walls, competing with antiferroelectric order (Krogstad *et al.*, 2018 ▸). The typical length scale of the disorder is of the order of 10 nm, deduced from the extent of the characteristic diffuse scattering observed using X-rays and neutrons (Krogstad *et al.*, 2018 ▸). Despite numerous studies, the connection between the structure, dynamics and outstanding materials properties remains elusive. As such, we are exploring the use of XPCS to reveal the response of the nanoscale structure as PMN is subject to an applied electric field.

We first present XPCS results from PMN under an applied transverse AC field that show strong field-induced responses in the two-time correlation function (TTCF) from the speckle in the diffuse scattering, even for relatively weak applied fields. We show that the speckle responses arise primarily from a shifting of the entire speckle pattern, rather than a change in the speckle arrangement. We find that the speckle-pattern shifts are correlated with motions of the Bragg peak. The experimentally observed TTCF can be explained quantitatively using the local tilting of the X-ray-illuminated sample volume. A weaker effect was found at lower incident X-ray intensities, suggesting that static fields from surface charging may be important to the large speckle response. We confirm the role of surface charging by studying a sample having a surface coated with carbon to eliminate static charging but maintain the transverse applied field, which showed no response. We then present a model for non-uniform static surface charging from the micrometre-scale X-ray beam, and for the electrostrictive response of the material to describe the combination of the static field from the charging and the dynamic applied field. This model qualitatively reproduces the direction, sign and magnitude of the tilting. Finally, we discuss the implications of these results for XPCS and nanodiffraction studies of insulating materials in general, under dynamic applied field or changing X-ray illumination.

## 2. Materials and methods

### 2.1. Crystal synthesis and preparation

**Figure 1:**
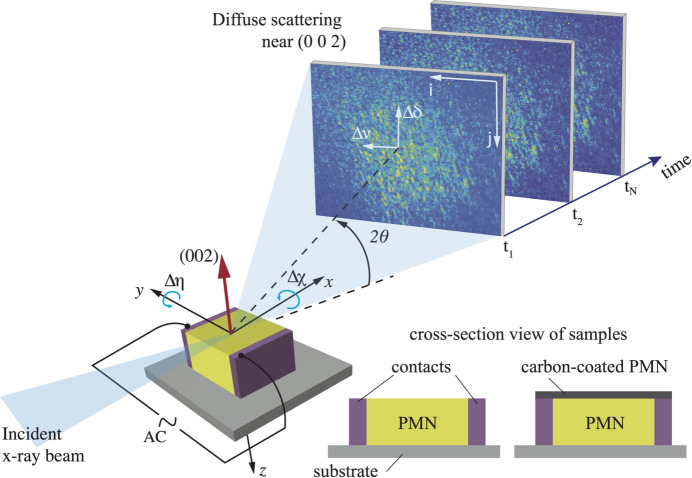
Experimental setup showing vertical, nearly symmetric-reflection scattering geometry and the coordinate axes. Insets: geometry of electrodes and carbon coating used on one sample.

The PMN single crystals were synthesized by a top-seeded solution growth method (Ye *et al.*, 1990 ▸). Their crystallographic orientations were determined using X-ray diffraction. Subsequently, the PMN crystals were cut and polished into cuboids with surface normals along the [0 0 1], [1 0 0] and [0 1 0] directions. The final surface finish was obtained using 1 µm grit size polishing compound. Silver epoxy electrodes were coated onto two opposite faces. The crystal was firmly mounted onto a sapphire substrate with epoxy resin to minimize current leakage and sample movement. Five electroded PMN crystals were fabricated. Unless otherwise stated, the data shown in this paper were collected from one crystal of size 0.97 mm × 0.56 mm × 0.49 mm in the *x*, *y* and *z* directions, respectively, shown in Fig. 1 ▸. The XPCS results obtained from all crystals were qualitatively consistent with the results presented in this paper.

### 2.2. Setup of the XPCS experiment

The XPCS measurements were performed in vacuum at room temperature in a wide-angle geometry using a standard four-circle diffractometer at Sector 8-ID-E of the Advanced Photon Source, USA. A schematic of the experimental setup and the scattering geometry is shown in Fig. 1 ▸. The incident X-ray beam was monochromated to a photon energy of 7.35 keV (wavelength λ = 1.687 Å). The source size was reduced horizontally using the white-beam slit and focused to obtain the desired coherence length with a 2D compound refractive lens. This gave almost equal vertical and horizontal beam sizes of *b* = 5 µm full width at half-maximum (FWHM) (Dufresne *et al.*, 2020 ▸). The unattenuated incident flux was 5.5 × 10^9^ photons s^−1^. A Lambda 250K area detector with a silicon sensor and a pixel size of *p* = 55 µm was placed at *R* = 1 m from the sample on the diffractometer arm to collect the speckle patterns. This set of parameters gave an ideal FWHM speckle size of ξ_id_ = λ*R*/(*pb*) = 0.61 pixels at the detector for the nearly symmetric-reflection scattering geometry used. Owing to aberrations in beamline optics, we expect the actual FWHM speckle size to be about twice this value, or ξ = 1.2 pixels, based on the measured speckle contrast.

PMN is cubic with a lattice constant of 4.04 Å. As shown in Fig. 1 ▸, the PMN sample was mounted with [1 0 0] and [0 1 0] directions aligned with the coordinates *x* and *y* of the sample surface, and the [0 0 1] surface normal opposite to the depth coordinate *z*. A sinusoidal electric field with a period of 100 s (0.01 Hz) was applied perpendicular to the scattering plane along the *y* direction using an Agilent 33522A function generator. The maximum peak-to-peak amplitude of the electric field applied was *E*_pp_ = 3.5 × 10^4^ V m^−1^, corresponding to a peak-to-peak voltage of 19.6 V from the function generator. The speckle patterns of the diffuse scattering near the (0 0 2) peak at a Bragg angle of 24.4° were collected with an exposure time of 0.5 s. These were subsequently binned by five exposures to give 2.5 s per binned image. The applied voltage was recorded for each speckle pattern. The minimum exposure time was limited by the available speckle intensity, and the frequency of the applied voltage was chosen accordingly to provide 40 binned images within each period.

## 3. Analysis and results

### 3.1. XPCS

**Figure 2:**
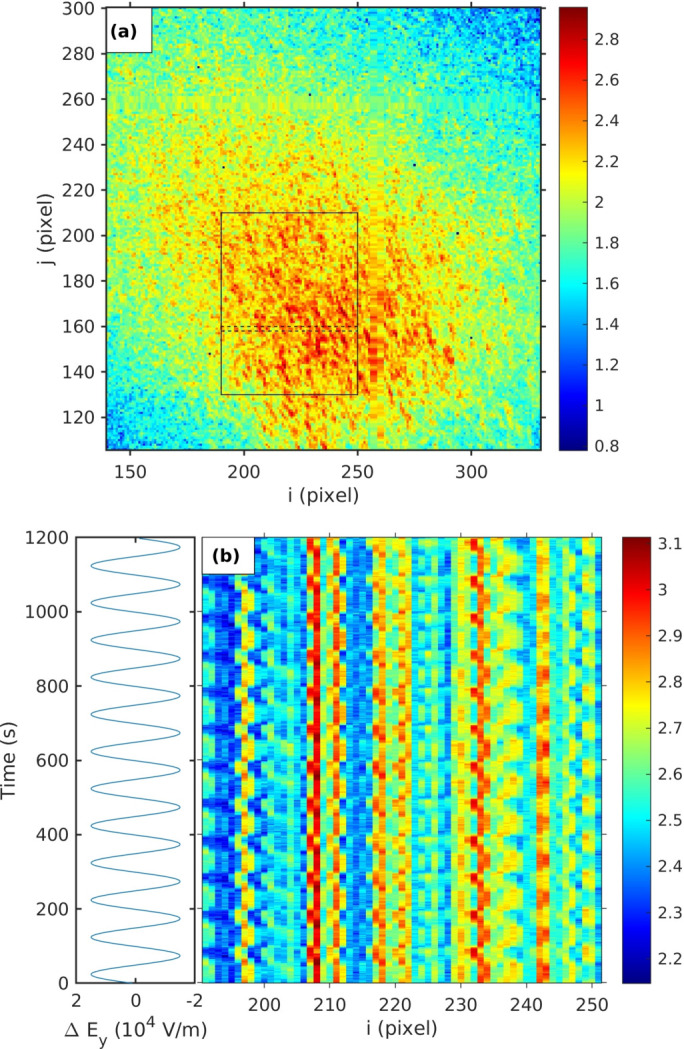
(*a*) Typical speckle pattern near (−0.02, 0.00, 1.98). The rectangle shows the region of interest used to calculate two-time correlations. (*b*) ‘Waterfall’ plot of the time dependence of the intensity in two rows of pixels shown by dashed lines in (*a*) during field cycling, given by a plot of Δ*E*_*y*_ versus time. The color scale is 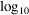 of counts in 2.5 s, per pixel (*a*) or per 2-pixel column sum (*b*).

Figure 2 ▸ shows a typical speckle pattern from the diffuse scattering near the (002) peak at *H* = −0.02, *K* = 0, *L* = 1.98. This is a single binned image at the beginning of a field cycle. This *HKL* position is selected because the diffuse scattering in PMN is most prominent along the {101}-type vectors away from Bragg peaks (Krogstad *et al.*, 2018 ▸), and this deviation from the (002) Bragg peak gave sufficient diffuse signal.

**Figure 3:**
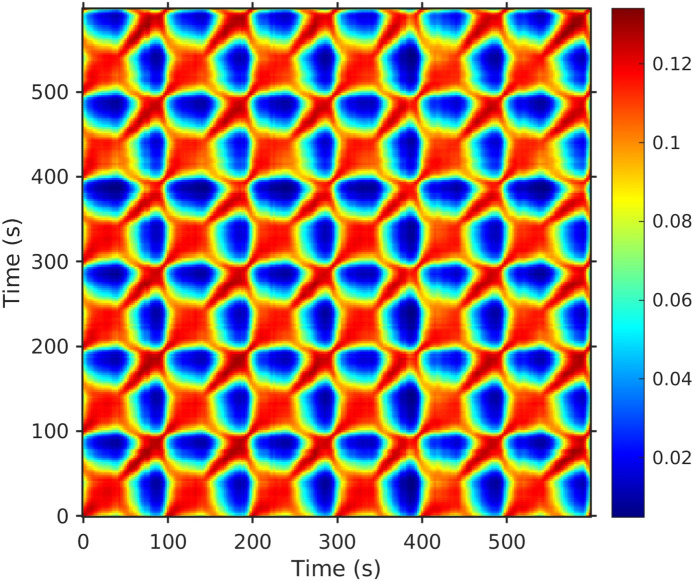
Typical two-time correlation function in the diffuse scattering over six field cycles, under a peak-to-peak electric field amplitude *E*_pp_ = 3 × 10^4^ V m^−1^ and an incident X-ray flux of *I*_tot_ = 5.5 × 10^9^ cps (photon counts per second). The color scale is the value of *C*.

To characterize the evolution of the speckle pattern as a function of applied field, we used a two-time correlation function (Bikondoa, 2017 ▸; Ju *et al.*, 2019 ▸) that accounts for the average intensity variation across the diffuse peak, and any potential variation of the average intensity with time. The correlation between speckle patterns acquired at times *t*_1_ and *t*_2_ was calculated using 

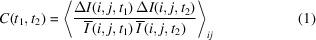

where 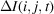 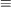 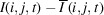 is the deviation of the speckle intensity from the average intensity 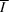 that would be measured under incoherent conditions where speckles are not resolved, and 〈…〉_*ij*_ denotes an average over all the pixels *ij* within the region of interest shown in Fig. 2 ▸. A two-dimensional Savitzky–Golay filter (Savitzky & Golay, 1964 ▸) was used to generate 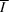 from the speckle intensity distribution at each time. In addition, the contribution from Poisson counting statistics 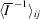 , where 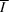 is expressed as the number of counts in the binned sum for each pixel, was subtracted from the diagonal elements at *t*_1_ = *t*_2_. Figure 3 ▸ shows a typical two-time correlation function over six voltage cycles. This exhibits strong correlations having the same period as the applied field and essentially no decay across many periods, indicating a highly reversible response. It is evident from the highly periodic pattern that all the dynamics are driven by the changing applied field. We verified that the sample had no observable equilibrium dynamics over these time scales by collecting datasets at constant applied field and confirming that they were static (constant *C*) over 1500 s.

**Figure 4:**
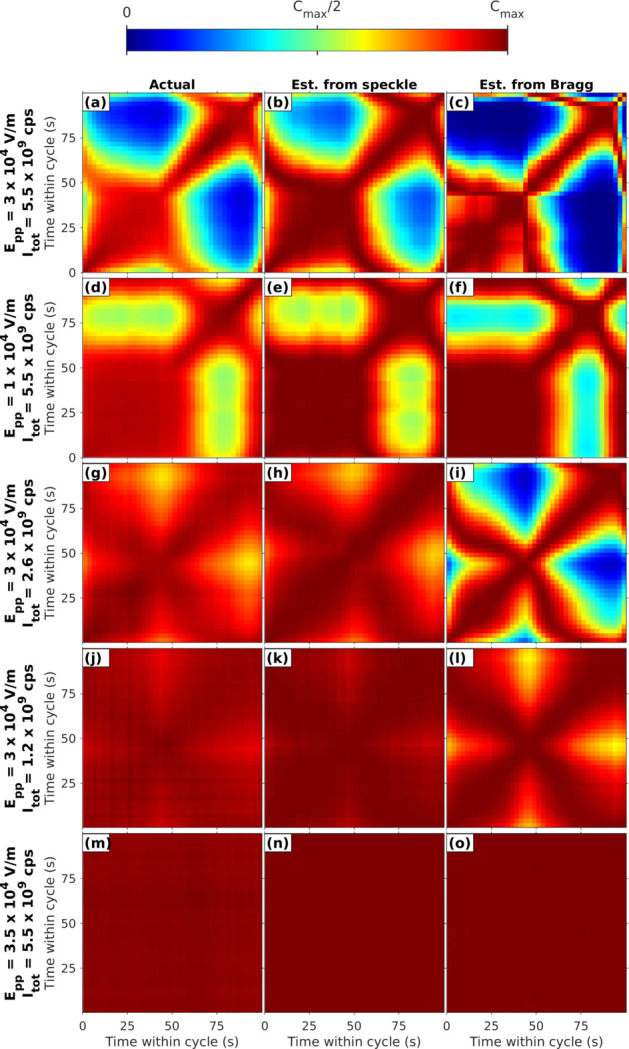
Two-time correlations within one cycle. Left column: actual measured from diffuse scattering in region shown in Fig. 2 ▸(*a*). Middle and right columns: estimated from speckle and Bragg motion. The first and second rows are for higher and lower applied field *E*_pp_ at high incident X-ray intensity *I*_tot_. Third and fourth rows are at reduced values of *I*_tot_ at higher *E*_pp_. The bottom row is for carbon-coated samples at high *E*_pp_ and *I*_tot_. The color scale ranges are set from zero to maximum of correlation given in Fig. S.3 of the supporting information.

Because of the strong periodicity, we focused our analysis on the behavior of *C*(*t*_1_, *t*_2_) within one period, calculated by summing *I*(*i*, *j*, *t*) over time points at the same position in the applied field cycle. Each dataset had 14 complete cycles [defined as starting with *t* = 0 at the first positive-going zero crossing of the applied field Δ*E*_*y*_(*t*) = 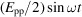 ], and we summed the final 12 cycles, to reduce any effects from initial transients. Figures 4 ▸(*a*) and 4(*d*) show such single-period two-time correlation functions for two values of the peak-to-peak field amplitude *E*_pp_. The response of the speckle pattern occurred for applied fields as small as *E*_pp_ = 5 × 10^3^ V m^−1^. The results presented here are based on an analysis of the region of diffuse scattering shown in Fig. 2 ▸(*a*), at an offset of −0.02 in the (1 0 1) direction from the (0 0 2) peak. We checked the dependence of the behavior on the location in reciprocal space, *i.e.* the offset from various Bragg peaks. Apart from changes in overall intensity, there was no qualitative change in the two-time behavior with *HKL*; all locations investigated [*e.g.* offset of −0.01, and (0 0 1) and (1 0 3) peaks] showed similar strong two-time correlations for the same applied field amplitude.

### 3.2. Speckle motion

Examination of the speckle patterns at different points in the field cycle indicated that the main change was a periodic shifting of the complete pattern on the detector, rather than a change in the arrangement of the speckles. Figure 2 ▸(*b*) shows a ‘waterfall’ plot of the intensity in two rows of pixels across the detector as a function of time, illustrating the periodic shifting. Such shifts can be analyzed by a form of ‘speckle tracking’ to obtain the local deformation that produces them (Sutton *et al.*, 2021 ▸). To extract the time-dependent amplitude of such shifts, we calculated a χ^2^ metric that characterized the similarity of the pattern at time *t*_1_ to the pattern at time *t*_2_ subject to a variable shift in pixels by Δ*i* and Δ*j*, 

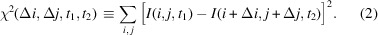

For each time pair (*t*_1_, *t*_2_), we located the values of Δ*i*, Δ*j* that minimize χ^2^. The best results were obtained by fitting 1/χ^2^ to a Gaussian peak with fixed σ of 2 pixels in *i* and *j*, and ignoring the central point at Δ*i* = Δ*j* = 0 which contains a contribution from systematic errors such as detector non-uniformity. This gives two-time matrices of the difference in speckle location between times *t*_1_ and *t*_2_ for each pixel direction Δ*i*_min_(*t*_1_, *t*_2_) and Δ*j*_min_(*t*_1_, *t*_2_) which are anti-symmetric in *t*_1_ and *t*_2_. Because of the fitting procedure, shifts can be determined with sub-pixel resolution. To the extent that these two-time correlations result simply from shifting the speckle pattern by some time-dependent amounts Δ*i*(*t*) or Δ*j*(*t*), each row of the matrix gives that function, offset by different starting values, and each column gives the negative of the function. Thus, the average functions Δ*i*(*t*) and Δ*j*(*t*) can be obtained by averaging Δ*i*_min_(*t*_1_, *t*_2_) and Δ*j*_min_(*t*_1_, *t*_2_) along columns. Typical results of this process are shown in Figs. S.1–S.2 of the supporting information.

**Figure 5:**
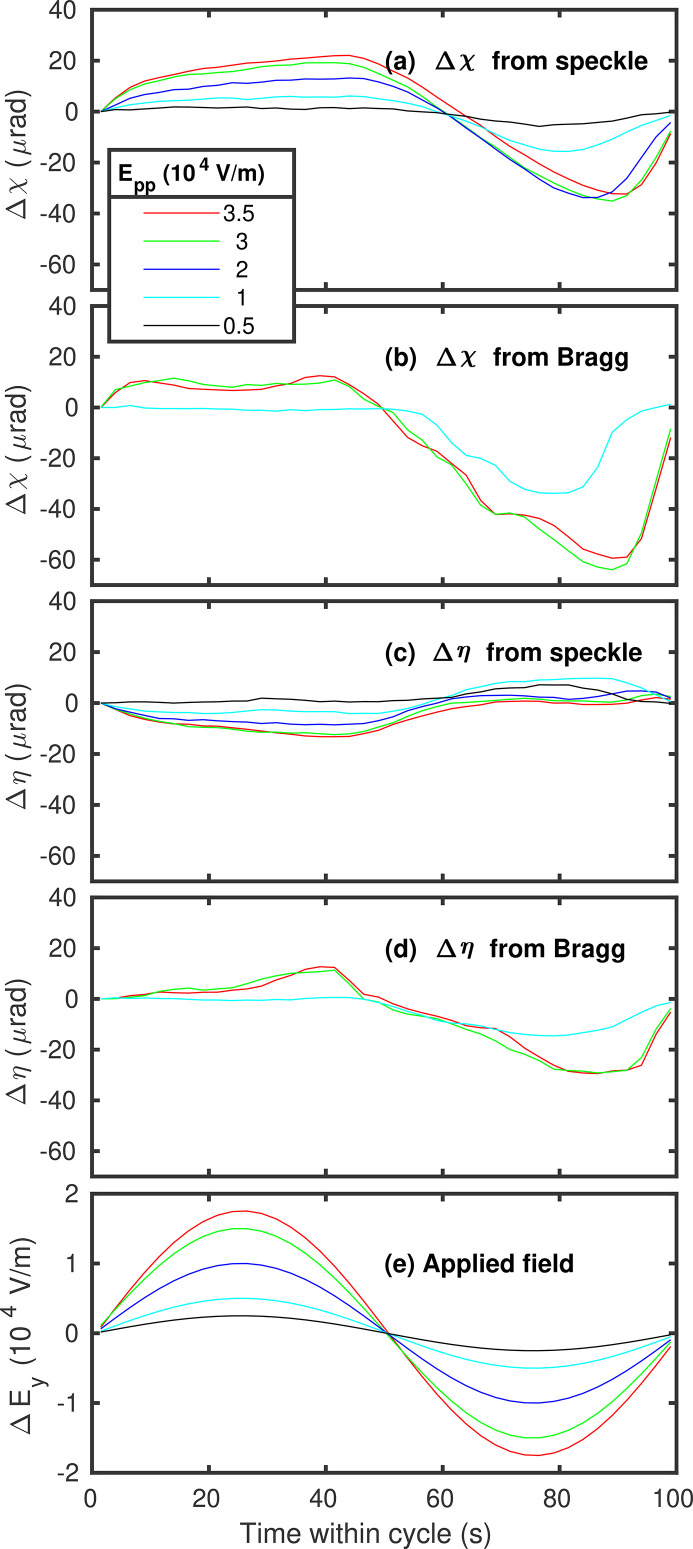
Measured change in sample angles determined from the speckle and Bragg peak motions as a function of time during a field cycle, for various field amplitudes. Incident beam intensity *I*_tot_ = 5.5 × 10^9^ cps.

**Figure 6:**
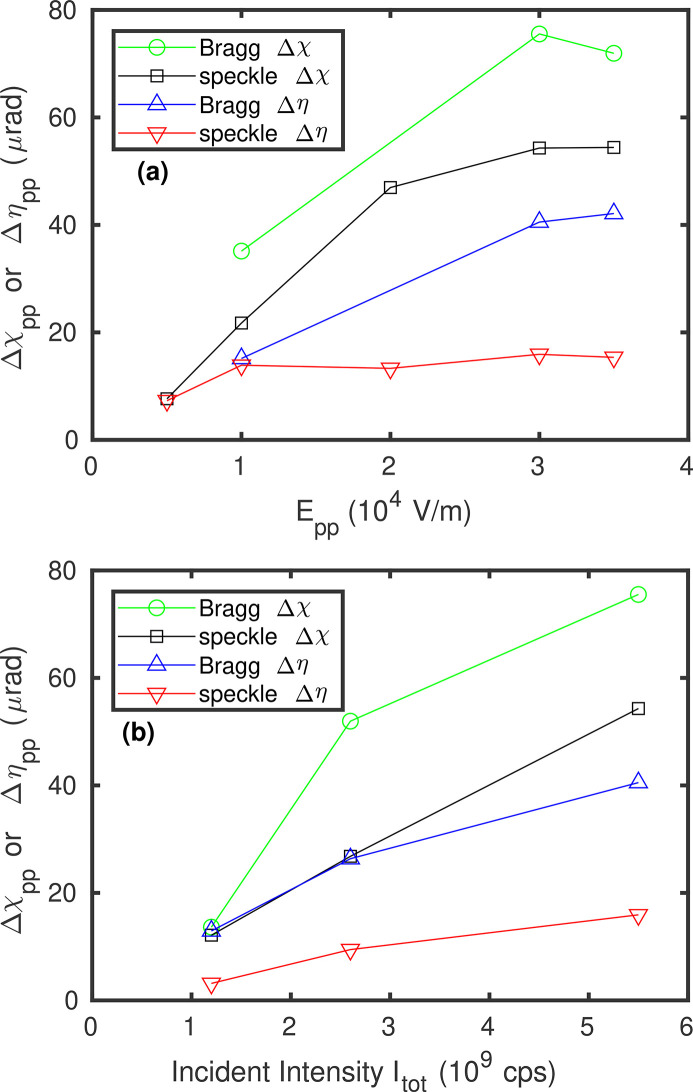
Peak-to-peak sample tilts in χ and η determined from the Bragg peak and speckle motions from Fig. 5 ▸, versus (*a*) the amplitude of the applied electric field at *I*_tot_ = 5.5 × 10^9^ cps and (*b*) the incident X-ray intensity at *E*_pp_ = 3 × 10^4^ V m^−1^.

These motions of the speckle pattern on the detector can be explained simply by a time-dependent tilting of the illuminated volume. For our geometry it is straightforward to convert the observed motion on the detector Δ*i*(*t*) and Δ*j*(*t*) into corresponding changes in the two sample angles Δη(*t*) and Δχ(*t*) shown in Fig. 1 ▸, using

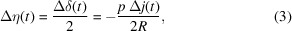




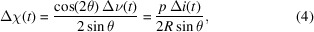

where θ is the Bragg angle. Figures 5 ▸(*a*) and 5(*c*) show the calculated sample angle changes during a field cycle from the observed speckle motion on the detector, for several field amplitudes. The amplitudes of the angle increase with the field amplitudes, as shown by the peak-to-peak tilt angles in Fig. 6 ▸(*a*). The angle changes are of the order of 50 µrad, larger in the χ direction than in the η direction.

We can estimate the two-time speckle correlations that would be expected from these sample angle changes, to check whether angle changes of this magnitude would explain the observed two-time correlation functions. As shown in the supporting information, time-dependent sample rotations Δχ(*t*) and Δη(*t*) give an estimated two-time correlation function of 

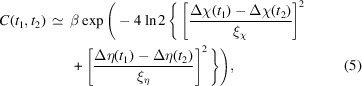

where the coherence factor 0 ≤ β ≤ 1 accounts for incomplete coherence of the incident beam and finite detector resolution, and we use normalized actual speckle sizes ξ_χ_ = 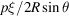 = 82 µrad and ξ_η_ = *p*ξ/2*R* = 34 µrad expressed as tilt angles. Although the tilts are larger in χ than in η, owing to the smaller ξ_η_ the speckle contrast is more sensitive to Δη, and so both contribute to the two-time behavior.

Figures 4 ▸(*b*) and 4(*e*) show the two-time correlation functions calculated using the sample angle changes determined from the speckle motion on the detector shown in Fig. 5 ▸. This agrees well with the observed two-time correlation functions of the speckle. Figure S.3(*a*) of the supporting information shows how the minimum and maximum values of the actual and estimated two-time correlation functions vary with applied field.

### 3.3. Bragg peak motion

If the hypothesis is correct that the speckle motion we observe in the diffuse scattering arises from a time-dependent tilt of the illuminated volume of the sample, we would expect to see corresponding motions of the Bragg peaks on the detector. We therefore recorded the scattering in the region of the (0 0 2) Bragg peak under the same applied field conditions as used for the diffuse scattering. We indeed observed motion of the peak, *i.e.* the intersection of the Bragg peak and its crystal truncation rod with the detector. We extracted the Bragg peak motion using the same analysis as described above for the motion of the speckle pattern, and converted it to sample angle tilts using the same expressions. The results are displayed in Figs. 5 ▸(*b*) and 5(*d*), for the same set of field amplitudes. The dependence of the peak-to-peak motion on field amplitude is given in Fig. 6 ▸(*a*). While the extracted motions of the Bragg peak are noisier than those of the speckle, the Δχ results are in reasonable agreement, with a similar amplitude, phase and field amplitude dependence. The Δη results from the Bragg motions have signs opposite to those from the speckle motions, although in both cases the angular changes are smaller in η than in χ. This difference is discussed in Section 6 below.

We can estimate the two-time correlation functions of the speckle from the sample angle changes extracted from the Bragg peak motion, just as we did for those from the speckle motion. These results are shown in Figs. 4 ▸(*c*) and 4(*f*). They also qualitatively reproduce the observed behavior, supporting the hypothesis of macroscopic tilting of the illuminated volume by the applied field.

## 4. Influence of X-ray illumination

It is remarkable that the small applied field used in our experiment can produce the observed tilting of more than 50 µrad. PMN is an electrostrictive material, where the induced strain depends quadratically on applied field, *e.g.* 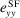 = 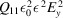 for a uniform field (see Section D of the supporting information). At zero field, the average polarization and linear piezoelectric response are zero. If the only contribution to the electric field is the applied field of Δ*E*_*y*_ = ±1.75 × 10^4^ V m^−1^, the calculated strain would be only ∼4 × 10^−7^, using expected values for the dielectric and electrostrictive constants (see supporting information). Furthermore, it is not obvious how a uniform field can produce a local rotation of the crystal orientation. We therefore investigated whether the X-ray illumination could affect the behavior, *e.g.* through the static electric field generated by local surface charging. Surface charging of insulating materials by X-ray illumination is a well known effect in X-ray photoelectron spectroscopy (Moulder *et al.*, 1992 ▸), and has been observed to shift photoelectron energies by >100 eV (Yasuno, 2019 ▸). In particular, small X-ray beams have been shown to produce local charging with non-uniform electric fields (Tielsch & Fulghum, 1996 ▸), potentially inducing tilting in the material.

Figures 4 ▸(*g*) and 4(*j*) show the two-time correlation functions from experiments in which we attenuated the incident beam by factors of 2.1 and 4.5. The variation in the correlation function is clearly reduced at lower incident beam intensities. Figure S.4 of the supporting information shows the measured sample tilts calculated from the observed speckle and Bragg peak motions for various incident X-ray intensities, with *E*_pp_ = 3 × 10^4^ V m^−1^. The tilts decrease at lower incident intensities, as shown by the peak-to-peak tilt ranges in Fig. 6 ▸(*b*). The two-time correlation functions estimated from the speckle and Bragg peak motions are shown in Figs. 4 ▸(*h*)–4(*i*) and 4(*k*)–4(*l*). Figure S.3(*b*) of the supporting information shows how the minimum and maximum values of the actual and estimated two-time correlation functions vary with incident beam attenuation. These results show a clear reduction in the response of the sample to the applied field when the incident X-ray intensity is reduced.

A standard method to reduce surface charging is to coat the surface with a thin conductive layer such as carbon (Suzuki *et al.*, 2020 ▸; Yang *et al.*, 2021 ▸). To experimentally test whether static charging of the sample surface by the X-ray beam could play a role, we prepared a different sample having a thin (∼1 nm) carbon film evaporated onto the surface, spanning the two electrodes, as shown in Fig. 1 ▸. This film geometry was designed to reduce or eliminate surface charging, while maintaining the same uniform applied field Δ*E*_*y*_. The measured electrical response of the sample after coating was consistent with a high resistance (*R* = 6 × 10^6^ Ω) added in parallel with the sample capacitance; before coating, the leakage was negligible (*R* > 10^9^ Ω). This sample had dimensions 0.84 mm, 0.57 mm and 0.49 mm in the *x*, *y* and *z* directions, respectively, shown in Fig. 1 ▸. Figure 4 ▸(*m*) shows the two-time correlation function for an applied field of *E*_pp_ = 3.5 × 10^4^ V m^−1^ with no incident beam attenuation. Figure S.5 of the supporting information shows the extracted tilt angles from the speckle and Bragg peak motions, and Figs. 4 ▸(*n*)–4(*o*) show the two-time correlation functions estimated from these motions. The carbon coating completely eliminated the response to the applied field, consistent with eliminating the effect of the static surface charge.

## 5. Model for surface charging and tilting

To explain the results presented above, our hypothesis is that the sample surface region has a steady-state electric field distribution caused by charging in the area of X-ray illumination owing to the ejection of photoelectrons. This static field, combined with the small dynamic applied field, results in local deformation from the electrostrictive properties of the sample, giving rise to the observed Bragg peak motion and two-time correlation functions of the speckle.

**Figure 7:**
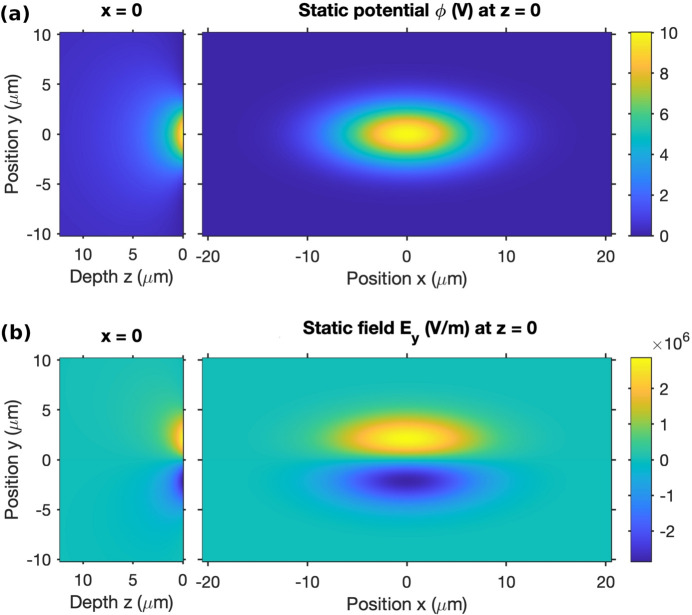
(*a*) Static potential ϕ and (*b*) electric field component *E*_*y*_ at the surface (right) and inside the sample (left).

We have calculated the potential and field distributions resulting from a Gaussian-shaped X-ray beam, using the experimental conditions. Details of the calculation are given in the supporting information. In summary, we assume that charging from X-ray absorption produces a surface potential proportional to the incident X-ray intensity, and solve Laplace’s equation to obtain the potential distribution inside the sample. Figure 7 ▸ shows the steady-state electric potential and transverse field distributions for an assumed peak static potential of ϕ_pk_ = 10 V. This gives a maximum value of the static in-plane electric field of *E*_*y*_ = 2.9 × 10^6^ V m^−1^. Because of the quadratic dependence of strain on field in the electrostrictive material, the large static field greatly increases the response to the small dynamic field. Furthermore, the peak potential ϕ_pk_ could be much higher than the 10 V assumed. While the rate of charging can be estimated fairly accurately, the rate of discharging due to leakage is unknown, so the steady-state peak potential ϕ_pk_ = 10 V is simply an assumed value. The potential could approach the escape voltage of the photoelectrons, which is several kilovolts. Thus surface charging to ϕ_pk_ > 1000 V is possible, which would give a static field *E*_*y*_ > 2.9 × 10^8^ V m^−1^.

We have also calculated the mechanical displacement and local tilting distributions due to a small, uniform, dynamic applied field in the *y* direction, Δ*E*_*y*_, in the presence of the static electric field distribution from the X-ray illumination. The spatial coordinates *x* and *y* in the plane of the surface and depth *z* into the surface are shown in Fig. 1 ▸. Details of the calculation are given in the supporting information. In summary, the displacement is related to the total strain, which is the sum of two terms: the stress-free strain from the electrostrictive response to the electric field, and the elastic strain that arises to make the total strain compatible with a displacement field and match the stress boundary conditions at the surface. We model PMN as a linear dielectric with electrostriction. While the effective dielectric constant has been found to decrease at higher fields (Hoover *et al.*, 1997 ▸), for simplicity we use a fixed dielectric constant. We discuss the limitations of this model below.

**Figure 8:**
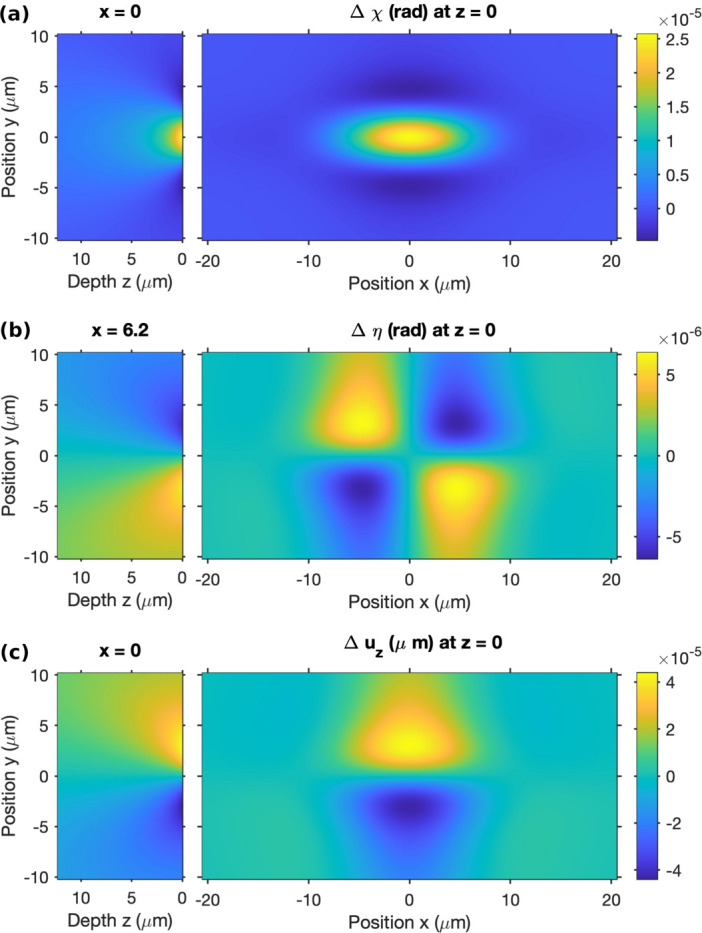
(*a*) Dynamic tilt Δχ, (*b*) dynamic tilt Δη and (*c*) dynamic displacement Δ*u*_*z*_ at the surface (right) and inside the sample (left) for Δ*E*_*y*_ = 1.75 × 10^4^ V m^−1^, ϕ_pk_ = 10 V.

With these assumptions, the model gives an exact solution for the strain response to the applied field, in the presence of the static field. One of its general predictions is that the tilt is largest in the χ direction. Figure 8 ▸ shows the calculated displacement and tilt distributions. The change in the tilt at the surface Δχ_0_ due to the dynamic applied field has maximum magnitude at the center of the illuminated region, which can be evaluated as 

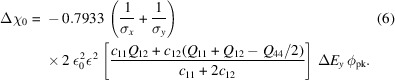

Here, σ_*x*_ and σ_*y*_ are the σ values of the Gaussian beam profile on the surface, while *c*_*ij*_, *Q*_*ij*_ and ε are the elastic, electrostrictive and dielectric constants. Our beam size of *b* = 5 µm at an incidence angle of θ_B_ = 24.4° gives σ_*x*_ = 5.1 µm and σ_*y*_ = 2.1 µm. For PMN, the quantity in square brackets has a value of −2.8 × 10^−3^ m^4^ C^−2^ and the value of ε_0_ε is 2.2 × 10^−7^ C m^−1^ V^−1^ (see supporting information). Using ϕ_pk_ = 10 V and a dynamic applied field amplitude *E*_pp_ = 3.5 × 10^4^ V m^−1^, the predicted peak–peak amplitude of Δχ_0_ is Δχ_pp_ = 5.2 × 10^−5^, in reasonable agreement with the observed values. Furthermore, the predicted sign of Δχ_0_ is positive for positive Δ*E*_*y*_, also in agreement with the observations. For the assumed symmetric beam profile, the predicted tilt in the η direction is zero at the center.

## 6. Discussion and conclusions

As described above, the strong periodic two-time correlation functions we observe in the speckle patterns from PMN under an applied AC field of modest amplitude arise from motions of the complete speckle pattern that are similar to motions of the Bragg peak. These can be interpreted as local tilting of the illuminated volume. Figure 4 ▸ shows that the two-time behavior estimated from the speckle pattern motion is in good agreement with that observed experimentally, while the somewhat larger tilts extracted from the Bragg peak motion give a higher estimated contrast. Figure 5 ▸ shows that the tilting grows almost linearly with both field and incident X-ray intensity, with some indication of saturation at higher values. A model based on static surface charging due to X-ray illumination and electrostrictive material response predicts these linear dependences, with a sign, direction and magnitude of the tilting that agree with the observations. While the initial goal of the study was to characterize the response of the nanoscale structure of PMN to the applied field, our XPCS results are dominated by the bulk electrostrictive response to X-ray charging, which would arise even if the nanoscale structure was otherwise unaffected by the small applied field.

As shown in Fig. S.2 of the supporting information, we typically observed that the extracted speckle motions in the η direction were noisier and less consistent with a simple overall time-dependent shift than those in the χ direction. Also, the direction of the η motion of the Bragg peak was opposite to that of the speckle, unlike the χ motion. These effects can be understood from the predicted in-plane distributions of the χ and η tilts shown in Figs. 8 ▸(*a*) and 8(*b*). While the χ tilt is primarily a single peak centered on the beam position, the η tilt is a complex pattern having lobes of opposite sign, and a value of zero at the center of the beam. Any asymmetry in the diffracting region, not included in the model, would give potentially complex behavior of the average η tilt. In particular, the Bragg diffraction could arise from a more non-uniform area than the diffuse scattering, owing to defects such as dislocations in the crystal.

We sometimes observed transients of a few hundred seconds before the two-time correlation function reached a steady-state periodic behavior, because X-ray exposure began at the start of each dataset. From our charging model, we estimate that the time to reach steady-state should typically be a few hundred seconds or less. We thus omitted the first two cycles from our analysis of each dataset.

There are two issues with the quantitative agreement between the experimentally observed tilts and those predicted by the model. The first is that the model does not account for any migration of charge in the sample, so the steady-state potential distribution is the same size as the X-ray intensity distribution. The calculated χ tilt averaged over the illuminated volume is significantly smaller than the peak tilt, *e.g.* 12 versus 52 µrad, and agrees less well with the experiment. However, if some charge migration were included in the model, then the steady-state potential distribution would be larger than the illuminated area, making the average tilt closer to the peak tilt. The reduction in static electric field because of the smaller gradient could be compensated by a larger peak potential, giving an equivalent static field and tilt over the larger area, since the charging effect of the X-rays could produce peak potentials much higher than the 10 V assumed.

The second issue is that the model is based on constitutive relations for a uniform electrostrictive medium with a uniform dielectric constant. It has been found that the bulk induced polarization of PMN saturates at a value of about *P*_sat_ = 0.3 C m^−2^ (Li *et al.*, 2014 ▸) at high fields, giving an effective dielectric constant that decreases with increasing field. In our model, we assume a dielectric constant fixed at the low-field value, so that the polarization continues to increase linearly as field increases. We can thus match the predicted tilts from this model to even the largest observed tilts, using literature values for electrostrictive and elastic coefficients, since the static field produced by the X-ray could have very large values. However, the case illustrated with ϕ_pk_ = 10 V and *E*_*y*_ = 2.9 × 10^6^ V m^−1^ already gives a calculated polarization of *P*_*y*_ = 0.64 C m^−2^, exceeding the saturation polarization.

A related issue is that the linear electrostrictive constitutive relations predict a tilt response that is in phase with the applied AC field. The results in Fig. 5 ▸ show that the observed tilt response tends to have a phase lag with respect to the applied field, which becomes more apparent for higher fields. Due to the large static field from the X-ray illumination, we speculate that the response to the small applied AC field may become hysteretic, *e.g.* from reorientation of nanoscale polarization domains. At lower temperatures, *e.g.* 210 K, a field-induced transformation to a hysteretic ferroelectric state is known to strongly influence the properties (Cowley *et al.*, 2011 ▸; Ye & Schmid, 1993 ▸). Such hysteresis, not included in the model, would tend to generate a phase lag of the maximum tilt with respect to the maximum applied field. It would also produce a distorted tilt versus time plot that is not a simple sinusoid, also in qualitative agreement with what we see. It is possible that hysteresis in micrometre-scale regions also gives a larger effective piezoresponse than predicted by the bulk-properties-based linear electrostrictive model, explaining the high apparent polarization values needed in the model.

Thus, the simple electrostrictive model, consistent with the overall cubic symmetry of bulk PMN and literature values for electrostrictive, elastic and dielectric coefficients (see supporting information), explains the main features of the measurements: it gives a tilt primarily in the χ direction of a magnitude and sign similar to that observed, that increases with applied field and with X-ray illumination intensity. These trends are clear in Fig. 6 ▸, and are consistent with the linear dependence on applied field and X-ray intensity predicted by equation (6). The deviations from the model, such as a saturation of the tilts as a function of applied field and X-ray intensity (rather than a continued linear increase), and the phase lag and non-sinusoidal response at higher applied field amplitudes, are the sort of deviations that would be expected from deviations of the response from simple electrostrictive behavior, *e.g.* because of saturation of the polarization and hysteretic response to the applied field. Likewise the smaller tilts observed in the η direction, not predicted by the model for a symmetric illuminated area, is a deviation that would be expected if the illumination is not perfectly symmetric.

Note that the scale of the displacement in Fig. 8 ▸(*c*) is in micrometres, so the maximum predicted displacement of ∼4 × 10^−5^ µm is less than 1 Å. The reason why XPCS is sensitive to such small displacements is because of their variation over micrometre distances, which produces tilt angles of tens of microradians.

Finally, we discuss the generality of the observed X-ray-induced effects. The model suggests that local tilts of 50 µrad or more, significant for XPCS, coherent diffraction imaging and high-resolution nanodiffraction, can occur whenever micrometre-scale, intense X-ray beams illuminate insulating crystals. While in our case we observed tilts in response to a dynamic applied field with static X-ray illumination, the model suggests that dynamic tilting can occur without applied field if the intensity of the X-ray illumination is varied, *e.g.* by scanning a small beam across a sample, or moving a small beam to a new location. Although the response of PMN is particularly large because of its high dielectric constant at room temperature, the elastic and electrostrictive constants are similar to those of many dielectric materials. For example, the commonly used crystal substrate SrTiO_3_ has a value for the combination of elastic and electrostrictive coefficients in brackets in equation (6) very similar to PMN, *i.e.* −2.5 × 10^−3^ m^4^ C^−2^ (Li *et al.*, 2006 ▸). Its dielectric constant at room temperature is 80 times smaller than PMN, but it grows to a similar value at low temperatures (Yang *et al.*, 2022 ▸). Even at room temperature similar X-ray-induced tilts would be possible since the peak potential could be 1000 times larger than assumed here, and the effect may reach saturation in PMN.

In summary, X-ray-induced surface charging, tilting and piezoresponse should be considered in designing XPCS and other high-resolution diffraction measurements using small intense X-ray beams. This may become especially important at new ultra-high-brightness X-ray sources such as the Advanced Photon Source after the upgrade. These effects can be mitigated by coating the surface with a conductive film or using a gas atmosphere to avoid charging; measurements in this study were made in vacuum. For scanning experiments, scanning either much more quickly or slowly than the time constant for charging could be an additional strategy.

## 7. Related literature

The following references, not cited in the main body of the paper, have been cited in the supporting information: Ahart *et al.* (2007 ▸); Bras *et al.* (2021 ▸); Griffiths (1999 ▸); Gullickson (2010 ▸); Hruszkewycz *et al.* (2012 ▸); Lee *et al.* (1999 ▸); Love (1944 ▸); Stephenson & Elder (2006 ▸); Uchino *et al.* (1980 ▸).

## Supplementary Material

Supporting information. DOI: 10.1107/S1600577523009116/vl5014sup1.pdf
