# Supplementary material for: X-ray-induced piezoresponse during X-ray photon correlation spectroscopy of PbMg1/3Nb2/3O3
Source: J Synchrotron Radiat. 2024 Jan 1;31(Pt 1):55–64. doi: 10.1107/S1600577523009116 (PMC10833419; doi:10.1107/S1600577523009116)
Supplement: Supplementary file 1 [file s-31-00055-sup1.pdf]

Supporting Information for "X-ray Induced Piezoresponse during X-ray Photon Correlation Spectroscopy of $PbMg_{1/3}Nb_{2/3}O_3$"

Dina Sheyfer,[1,2,*] Hao Zheng,[1,*] Matthew Krogstad,[1] Carol Thompson,[3] Hoydoo You,[1] Jeffrey A Eastman,[1] Yuzi Liu,[4] Bi-Xia Wang,[5] Zuo-Guang Ye,[5] Stephan Rosenkranz,[1] Daniel Phelan,[1] Eric M. Dufresne,[2] G. Brian Stephenson,[1,†] and Yue Cao[1,‡]

[1] *Materials Science Division; Argonne National Laboratory; Lemont IL 60439 USA*
[2] *X-ray Science Division; Argonne National Laboratory; Lemont IL 60439 USA*
[3] *Department of Physics; Northern Illinois University; DeKalb IL 60115 USA*
[4] *Center for Nanoscale Materials; Argonne National Laboratory; Lemont IL 60439 USA*
[5] *Department of Chemistry and 4D Labs; Simon Fraser University; Burnaby BC V5A 1S6, Canada*

This Supporting Information for a study of X-ray-induced piezoresponse during XPCS of $PbMg_{1/3}Nb_{2/3}O_3$ (PMN) under applied AC field includes examples of speckle motion extraction, estimation of two-time correlations from these motions, supplemental figures of results, and derivations of models for the surface charging due to X-ray illumination and for surface tilting due to electrostriction.

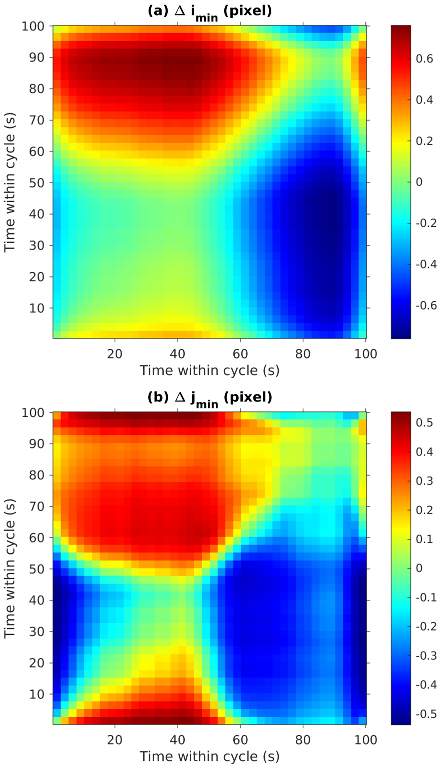

Figure S.1 Extracted two-time matrices in speckle shifts in (a) Δi and (b) Δj, for applied field $E_{pp} = 3 \times 10^4$ V/m and incident X-ray intensity $I_{tot} = 5.5 \times 10^9$ cps. Shifts in pixels are proportional to shifts in ν or δ, with one pixel in i or j corresponding to 84 or 55 μrad in ν or δ, respectively.

1. SPECKLE MOTION SUPPLEMENTAL FIGURES

Figure S.1 shows examples of the two-time matrices of the shifts in pixels $\Delta i_{min}(t_1,t_2)$ and $\Delta j_{min}(t_1,t_2)$ that minimize the difference between speckle patterns at two times in the field cycle. Figure S.2(a) and (b) show the values from each row of these matrices. The curves in Fig. S.2(a) all have similar shapes, consistent with the extracted differences of all time pairs $\Delta i_{min}(t_1,t_2)$ arising from the average time-dependent shift $\Delta i(t)$ shown in Fig. S.2(c). The curves shown in Fig. S.2(b) are noisier and have different shapes. Thus the extracted pair differences in this direction are only approximately attributable to the average time-dependent shift $\Delta j(t)$ shown in Fig. S.2(c).

2. ESTIMATING TWO-TIME CORRELATIONS FROM SAMPLE TILTING

We wish to estimate the two-time correlation of the speckle pattern arising from sample tilts $\Delta\chi(t)$ and $\Delta\eta(t)$ determined from observed speckle motions on the detector of $\Delta i(t)$ and $\Delta j(t)$. This is conveniently done by considering the reciprocal space coordinates $Q_h = (2\pi/a)H$, $Q_k = (2\pi/a)K$, and $Q_\ell = (2\pi/a)L$, where $a = 4.04$ Å is the lattice constant of cubic PMN. Sample tilts $\Delta\eta$ and $\Delta\chi$ will produce motions at a given point on the detector of the sample-fixed reciprocal space of

$$\Delta Q_h(t) = \frac{4\pi\sin\theta\,\Delta\eta(t)}{\lambda} = -\frac{2\pi p\sin\theta\,\Delta j(t)}{\lambda R}, \quad \text{(S.1)}$$

$$\Delta Q_k(t) = \frac{4\pi\sin\theta\,\Delta\chi(t)}{\lambda} = \frac{2\pi p\,\Delta i(t)}{\lambda R}, \quad \text{(S.2)}$$

$$\Delta Q_\ell(t) = \frac{4\pi\cos\theta\,\Delta\eta(t)}{\lambda} = -\frac{2\pi p\cos\theta\,\Delta j}{\lambda R}. \quad \text{(S.3)}$$

If the sample is illuminated with a coherent incident X-ray beam of size b and absorption length ℓ_{abs} in symmetric reflection geometry, the ideal FWHM speckle sizes in reciprocal

* These authors contributed equally.
† Correspondence to: gbs@anl.gov
‡ Correspondence to: yue.cao@anl.gov

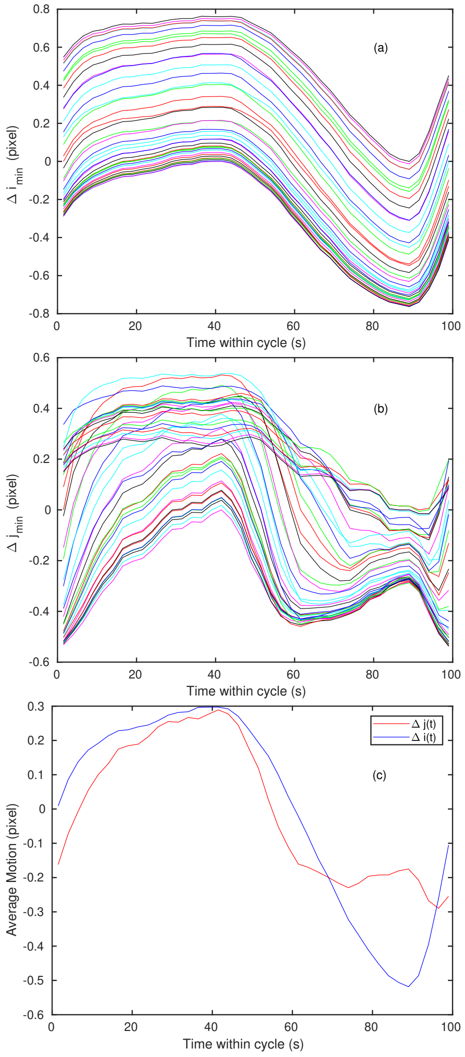

Figure S.2 (a) and (b) Estimates of speckle pattern shifts $\Delta i(t)$ and $\Delta j(t)$ from each row of matrices in Fig. S.1. (c) Average shifts $\Delta i(t)$ and $\Delta j(t)$ obtained by summing columns of matrices.

space will be (Hruszkewycz *et al.*, 2012)

$$\xi_{Qh}^{id} = \frac{2\pi \sin\theta}{b}, \tag{S.4}$$

$$\xi_{Qk}^{id} = \frac{2\pi}{b}, \tag{S.5}$$

$$\xi_{Q\ell}^{id} = \frac{4\pi}{\ell_{abs}\sin\theta}. \tag{S.6}$$

If we assume the same ratio between the actual and ideal ξ_{Qh} and ξ_{Qk} that we use for the speckle size on the detector ξ, then we can write the actual FWHM speckle sizes in Q and in tilt angles as

$$\xi_{Qh} = \frac{4\pi \sin\theta\, \xi_\eta}{\lambda} = -\frac{2\pi p \sin\theta\, \xi}{\lambda R}, \tag{S.7}$$

$$\xi_{Qk} = \frac{4\pi \sin\theta\, \xi_\chi}{\lambda} = \frac{2\pi p\, \xi}{\lambda R}. \tag{S.8}$$

The time-dependent changes ΔQ_i due to the sample rotations give an estimated two-time correlation function of the speckle of

$$\begin{aligned} C(t_1,t_2) &\approx \beta \exp\left(-4\ln 2 \sum_{m=h,k,\ell}\left[\frac{\Delta Q_m(t_1)-\Delta Q_m(t_2)}{\xi_{Qm}}\right]^2\right) \\ &\approx \beta \exp\left(-4\ln 2\left[\left(\frac{\Delta\chi(t_1)-\Delta\chi(t_2)}{\xi_\chi}\right)^2 + \left(\frac{\Delta\eta(t_1)-\Delta\eta(t_2)}{\xi_\eta}\right)^2\right]\right), \\ &\approx \beta \exp\left(-4\ln 2\left[\left(\frac{\Delta i(t_1)-\Delta i(t_2)}{\xi}\right)^2 + \left(\frac{\Delta j(t_1)-\Delta j(t_2)}{\xi}\right)^2\right]\right), \end{aligned} \tag{S.9}$$

where we have introduced the coherence factor $0 \le \beta \le 1$ to account for incomplete coherence of the incident beam and finite detector resolution. In the second and third lines, we show that the same result is obtained if we ratio the tilts $\Delta\chi$ and $\Delta\eta$ to the speckle sizes normalized to the tilt angles $\xi_\chi = 82\,\mu$rad and $\xi_\eta = 34\,\mu$rad, respectively, or if we ratio the speckle motions Δi and Δj to the actual speckle size on the detector of $\xi = 1.2$ pixels, neglecting the ΔQ_ℓ term under the assumption that the illuminated volume is very thin in the z direction due to a short absorption length so that $\xi_{Q\ell}$ is very large.

3. SUPPLEMENTAL RESULTS FIGURES

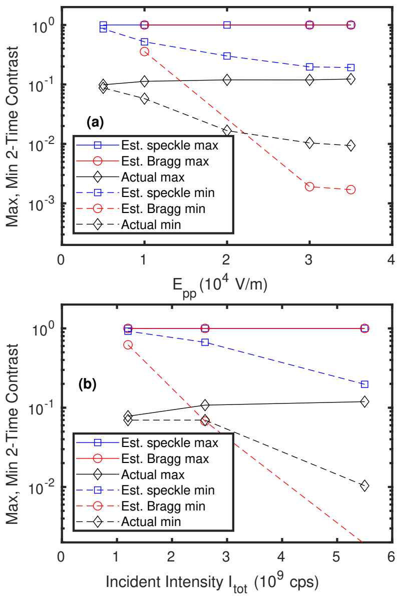

Figure S.3 Maximum and minimum two-time correlation values (actual and estimated from Bragg and speckle motions) vs. (a) applied field at $I_{tot} = 5.5 \times 10^9$ cps, (b) incident X-ray intensity at $E_{pp} = 3 \times 10^4$ V/m. For estimated values, maximum contrast β is set to unity to provide offset from actual.

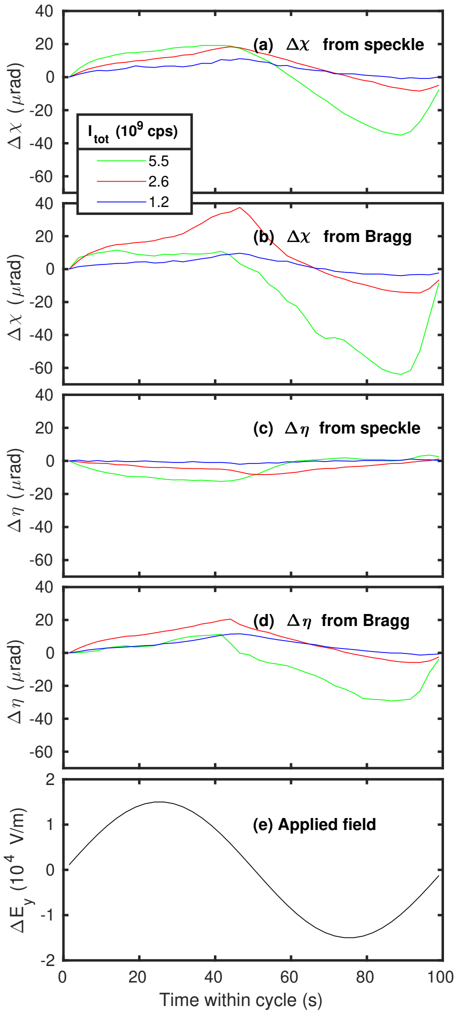

Figure S.4 Measured change in sample angles determined from speckle and Bragg motions as a function of time during field cycle, for different incident X-ray intensities. Peak-to-peak applied field was $E_{pp} = 3 \times 10^4$ V/m.

4. MODEL FOR DYNAMIC SURFACE TILTING FROM SMALL APPLIED FIELDS

Our hypothesis is that the sample has a steady-state electric field distribution caused by surface charging in the area of X-ray illumination owing to ejection of photoelectrons. This static field, combined with the small dynamic applied field, results in local deformation from the electrostrictive properties of the sample, giving the observed Bragg peak motion and two-time correlation functions of the speckle.

A. Surface Charging by X rays

Surface charging of insulating materials by X-ray illumination is a well-known effect in X-ray photoelectron spectroscopy (Moulder *et al.*, 1992), and has been observed to shift photoelectron energies by >100 eV (Yasuno, 2019). In

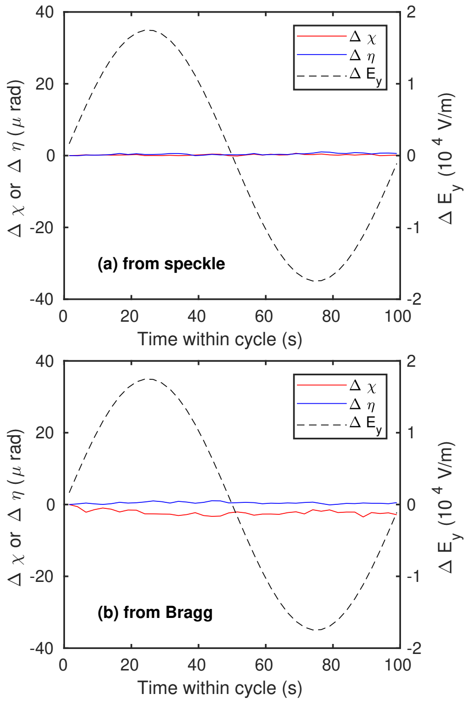

Figure S.5 Measured change in sample angles determined from (a) speckle and (b) Bragg motions as a function of time during field cycle, for carbon-coated sample. Peak-to-peak applied field was $E_{pp} = 3.5 \times 10^4$ V/m, incident X-ray intensity was $I_{tot} = 5.5 \times 10^9$ cps.

particular, small X-ray beams have been shown to produce local charging with non-uniform electric fields (Tielsch and Fulghum, 1996), potentially inducing tilting in the material. The first step in modeling this behavior is to calculate the steady-state electric potential and field distributions from X-ray-induced charging of the sample surface. For an insulating sample, X-ray illumination can result in surface charging because photoelectrons are ejected from the surface region. We assume that the photoelectron escape depth ℓ_{esc} is small, of the order of 10 nm (Bras *et al.*, 2021), and that the local free charge density is concentrated at the surface in a layer having a negligible thickness δz. One can estimate the slow dynamics and steady-state behavior of the surface charge density $\sigma(x,y)$ by writing a continuity equation for its rate of change,

$$\frac{d\sigma(x,y)}{dt} = -J_{out}(x,y) + J_{in}(x,y), \tag{S.10}$$

where the electric current densities J_{out} and J_{in} are the net photoelectric current density leaving the surface and the leakage current density back to the surface, respectively. These are all considered to be functions of the spatial coordinates x and y in the plane of the surface.

We assume that the photoelectric current density is proportional to the absorption rate of X rays in the surface region. For simplicity we assume that each photon produces a single photoelectron with kinetic energy sufficient to escape the charged surface. We can write the net photoelectric current

density as

$$J_{out}(x,y) = -e I_0(x,y) \left(\frac{\ell_{esc}}{\ell_{abs} \sin\theta_B + \ell_{esc}} \right) \tag{S.11}$$

where $I_0(x,y)$ is the distribution of incident X-ray intensity (counts per second per unit surface area), $-e$ is the electron charge, ℓ_{abs} is the X-ray absorption length, and θ_B is the incidence angle of the X rays. The quantity in parentheses gives the fraction of X rays absorbed within one photoelectron escape depth of the surface. (Here we neglect both the possibility of secondary electrons escaping, as well as the cutoff in net photoelectric current that will occur if the surface potential approaches the photoelectron energy.) We assume the leakage current density is proportional to the potential $\phi(x,y,0)$ at the surface $z=0$,

$$J_{in}(x,y) = \frac{-\phi(x,y,0)}{R_{leak}}, \tag{S.12}$$

where R_{leak} is the inverse of the effective conductance per unit area of the leakage medium, which could be the sample or the vapor environment.

The final relationship between the surface charge and potential is based on the Poisson equation relating surface charge and surface potential,

$$\sigma(x,y) = -\varepsilon_0 \, \varepsilon_s \, \delta z \, \nabla^2_{xy} \phi(x,y,0), \tag{S.13}$$

where $\varepsilon_0 = 8.85 \times 10^{-12}$ C V^{-1} m^{-1} is the permittivity of free space, and ε_s is the effective surface dielectric constant.

We can find a solution to these equations by expressing the surface potential $\phi(x,y,0)$ and the incident X-ray illumination $I_0(x,y)$ as two-dimensional Fourier series,

$$\phi(x,y,0) = \sum_{m=-\infty}^{\infty} \sum_{n=-\infty}^{\infty} \hat{\phi}_{mn}(0) \exp(iq_{xm}x) \exp(iq_{yn}y), \tag{S.14}$$

$$I_0(x,y) = \sum_{m=-\infty}^{\infty} \sum_{n=-\infty}^{\infty} \hat{I}_{0_{mn}} \exp(iq_{xm}x) \exp(iq_{yn}y), \tag{S.15}$$

where $\hat{\phi}_{mn}(0)$ and $\hat{I}_{0_{mn}}$ are the Fourier coefficients for wavenumbers $q_{xm} \equiv m\pi/a_x$ and $q_{yn} \equiv n\pi/a_y$, and $2a_x$ and $2a_y$ are the periods in the x and y directions, respectively. (To insure that the periodic boundary conditions in x and y do not affect the results, we use values of a_x and a_y much larger than the illuminated area.) Since ϕ and I_0 have real values, the Fourier coefficients for negative m and n are the complex conjugates of those for positive m and n. The in-plane Laplacian of the surface potential is given by

$$\nabla^2_{xy}\phi(x,y,0) = - \sum_{m=-\infty}^{\infty} \sum_{n=-\infty}^{\infty} (q^2_{xm} + q^2_{yn}) \, \hat{\phi}_{mn}(0) \exp(iq_{xm}x) \exp(iq_{yn}y). \tag{S.16}$$

Substituting Eqs. (S.11)-(S.16) into the continuity equation (S.10) gives a simple relaxation equation for the Fourier components of the surface potential

$$\frac{d\hat{\phi}_{mn}(0)}{dt} = \frac{\hat{\phi}^{ss}_{mn} - \hat{\phi}_{mn}(0)}{\tau_{mn}}, \tag{S.17}$$

where the steady-state surface potential Fourier coefficients $\hat{\phi}^{ss}_{mn}$ are given by

$$\hat{\phi}^{ss}_{mn} = B \hat{I}_{0_{mn}}, \tag{S.18}$$

$$B \equiv \frac{e R_{leak} \, \ell_{esc}}{\ell_{abs} \sin\theta_B + \ell_{esc}}, \tag{S.19}$$

and the relaxation time of each Fourier coefficient is

$$\tau_{mn} = R_{leak} \, \varepsilon_0 \, \varepsilon_s \, \delta z \, (q^2_{xm} + q^2_{yn}). \tag{S.20}$$

Thus the steady-state surface potential distribution is simply proportional to the incident X-ray intensity distribution,

$$\phi^{ss}(x,y) = B I_0(x,y). \tag{S.21}$$

We can solve for the three-dimensional static potential and field distributions inside the sample, assuming that the sample is an insulator with no free charge density. We assume that the relation between the polarization $\mathbf{P}$ and field $\mathbf{E}$ is isotropic. Then the Maxwell equation $0 = \nabla \cdot \mathbf{D} = \varepsilon_0 \nabla \cdot \mathbf{E} + \nabla \cdot \mathbf{P}$ is satisfied by a field distribution with $\nabla \cdot \mathbf{E} = 0$. This implies that the potential $\phi(x,y,z)$ obeys Laplace's equation, $\nabla^2 \phi = 0$, with the boundary condition of a specified surface potential $\phi(x,y,0)$. This can be solved using separation of variables (Griffiths, 1999), assuming ϕ is the sum of terms obeying Laplace's equation that are each products of functions in the x, y, and z directions. The three-dimensional potential that obeys Laplace's equation with the correct surface potential is then

$$\phi(x,y,z) = \sum_{m=-\infty}^{\infty} \sum_{n=-\infty}^{\infty} \hat{\phi}_{mn}(z) \exp(iq_{xm}x) \exp(iq_{yn}y), \tag{S.22}$$

with z-dependent Fourier coefficients for the x and y directions

$$\hat{\phi}_{mn}(z) = \hat{\phi}_{mn}(0) \exp[-(|q_{xm}| + |q_{yn}|)z]. \tag{S.23}$$

Here we assume that z is the depth into the sample, so that the sample extends in the positive z direction from the surface at $z=0$.

B. Steady-State Surface Charge

For the rest of this section we will assume that the surface potential has reached it steady-state value, $\phi(x,y,0) = \phi^{ss}(x,y)$. The x, y, and z components of the electric field vector $\mathbf{E} = -\nabla\phi$ can be written using expressions analogous to Eq. (S.22), with Fourier coefficients

$$\hat{E}_{xmn}(z) = -i q_{xm} \hat{\phi^{ss}}_{mn} \exp[-(|q_{xm}| + |q_{yn}|)z], \tag{S.24}$$

$$\hat{E}_{ymn}(z) = -i q_{yn} \hat{\phi^{ss}}_{mn} \exp[-(|q_{xm}| + |q_{yn}|)z], \tag{S.25}$$

$$\hat{E}_{zmn}(z) = (|q_{xm}| + |q_{yn}|) \hat{\phi^{ss}}_{mn} \exp[-(|q_{xm}| + |q_{yn}|)z]. \tag{S.26}$$

If we assume a form for the X-ray illumination $I_0(x,y)$, we can calculate the potential and field distributions. Here we

Table S.1 Parameter values used in calculating steady-state potential and field due to X-ray illumination.

$I_{tot} = 5.5 \times 10^9$ s^{-1}	$\ell_{esc} = 1 \times 10^{-8}$ m
$\sigma_y = 2.12 \times 10^{-6}$ m	$\ell_{abs} = 5.7 \times 10^{-6}$ m
$\sigma_x = 5.13 \times 10^{-6}$ m	$\theta_B = 24.4°$
$R_{leak} = 1.8 \times 10^2$ Ω m^2	$\phi_{pk} = 10$ V

assume that the illumination is the product of Gaussian functions in x and y with sigmas of σ_x and σ_y and a peak intensity density of I_{peak},

$$I_0(x,y) = I_{pk} \exp\left(\frac{-x^2}{2\sigma_x^2}\right) \exp\left(\frac{-y^2}{2\sigma_y^2}\right). \tag{S.27}$$

The integrated intensity in photons per second is

$$I_{tot} = 2\pi\sigma_x\sigma_y I_{pk}. \tag{S.28}$$

The Fourier coefficients of the intensity $I_0(x,y)$ and steady-state surface potential $\phi^{ss}(x,y)$ are

$$\hat{I}_{0_{mn}} = \frac{\pi I_{pk}\sigma_x\sigma_y}{2a_x a_y} \exp(-q_{xm}^2\sigma_x^2/2)\exp(-q_{yn}^2\sigma_y^2/2), \tag{S.29}$$

$$\hat{\phi}^{ss}_{mn} = \frac{\pi\phi_{pk}\sigma_x\sigma_y}{2a_x a_y} \exp(-q_{xm}^2\sigma_x^2/2)\exp(-q_{yn}^2\sigma_y^2/2), \tag{S.30}$$

where $\phi_{pk} = B I_{pk}$ is the maximum potential at the surface.

Figure S.6 shows the potential and field calculated using the parameter values in Table S.1. For a symmetric reflection geometry, we expect the width of the beam footprint to be longer in the scattering plane (x) than perpendicular to the scattering plane (y) by a factor of $1/\sin\theta_B$. Using the experimental beam size of 5 μm FWHM and $\theta_B = 24.4°$ gives $\sigma_y = 2.12\,\mu$m and $\sigma_x = 5.13\,\mu$m. For the calculations we use Fourier half-periods $a_x = 12\sigma_x$ and $a_y = 12\sigma_y$ and Fourier components in the range -24 to 24 for both m and n.

While the values of I_{tot}, σ_y, σ_x, and θ_B were determined in the experiments, and the absorption length ℓ_{abs} is straightforward to calculate (Gullickson, 2010) for PMN at the photon energy of 7.35 keV, the photoelectron escape length ℓ_{esc} is an estimate, and nothing is known about the value of R_{leak}. It was simply chosen to give a peak surface potential $\phi_{pk} = 10$ V.

For the conditions modeled, the maximum value of the steady-state in-plane electric field is about $E_y = 2.9 \times 10^6$ V m^{-1}. This is much higher than the maximum applied field, and so will greatly increase the electrostrictive strain. Furthermore, the peak potential ϕ_{pk} could be much higher than the 10 V assumed, since the value of R_{leak} in Eq. (S.12) is unknown. The potential could approach the escape voltage of the photoelectrons. For a photon energy of 7.4 keV, the primary absorption edge is Pb M5 at 2.5 keV, giving a primary photoelectron energy of 4.9 keV. Thus surface charging to $\phi_{pk} > 1000$ V is possible. Since E_y is simply proportional to ϕ_{pk}, this would give $E_y > 2.9 \times 10^8$ V m^{-1}.

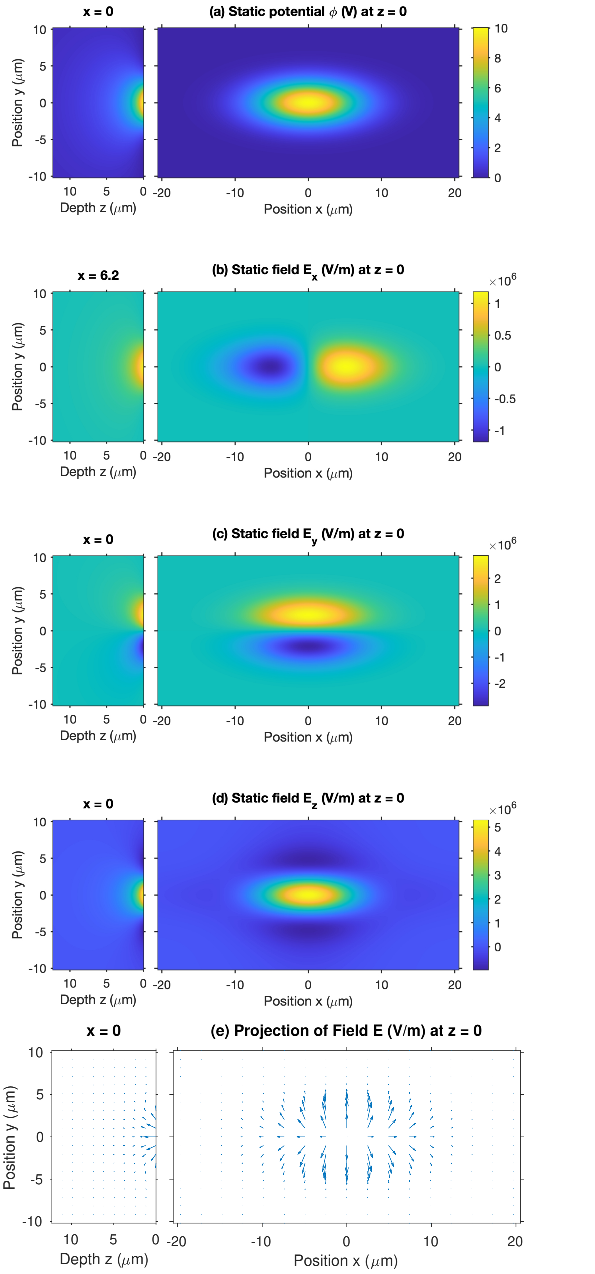

Figure S.6 Steady-state potential ϕ and electric field components E_x, E_y, and E_z at surface (right) and inside sample (left) using $\phi_{pk} = 10$ V.

C. Time to Establish Steady-State

An estimate of the typical time needed to establish the steady-state field can be obtained from Eq. (S.20) using the parameters in Table S.1 by substituting in the typical wavenumbers $q_{xm} = 2\pi/\sigma_x$, $q_{yn} = 2\pi/\sigma_y$. If we use the bulk dielectric constant $\varepsilon = 2.5 \times 10^4$ for PMN for the surface dielectric constant ε_s and a surface layer thickness δz equal to the photoelectron escape depth ℓ_{esc}, then the typical time constant is about 4 s. Another estimate can be obtained by con-

sidering the time it takes for the surface charge σ to reach the saturation polarization for PMN, $P_{sat} \approx 0.3$ C m^{-1}. Using a maximum surface charging rate of

$$-J_{out}(0,0) = \frac{e\,I_{tot}}{2\pi\sigma_x\sigma_y}\left(\frac{\ell_{esc}}{\ell_{abs}\sin\theta_B + \ell_{esc}}\right) \tag{S.31}$$

gives a charging time $\tau_{sat} \equiv -P_{sat}\,/\,J_{out}(0,0) \approx 5$ s.

D. Surface Tilting due to Electrostriction

In this section we calculate the displacement field and local tilting due to a small, uniform, dynamic applied field in the y direction, ΔE_y, in the presence of the static field distribution from the X-ray illumination, calculated above. The displacement field is related to the total strain, which in turn is the sum of the stress-free strain from the electrostrictive response to the electric field, and the elastic strain that arises to make the total strain compatible with a displacement field and to match the stress boundary conditions. We first obtain relations for the change in the stress-free strain components as a function of ΔE_y. We then use an *ansatz* to obtain the change in the displacement field that satisfies the required strain and stress conditions. The local tilts resulting from this changing displacement field can then be calculated. The relations between the strain, stress, and displacement components are linear, so the changes in these quantities due to the dynamic applied field have the same relationships as the total quantities.

We model the PMN as a linear dielectric with electrostriction. For small fields, the polarization components are proportional to electric field,

$$\mathbf{P} = \varepsilon_0\varepsilon\,\mathbf{E}, \tag{S.32}$$

where ε is the relative dielectric constant. This linearity is known to break down for fields above about 5×10^5 V m$^-1$ (Hoover *et al.*, 1997), as the effective dielectric constant decreases with field and the polarization saturates and becomes hysteretic. For simplicity, we will use the linear relationship Eq. (S.32), and discuss the limitations of this model elsewhere.

The stress-free strain components are quadratically related to polarization by (Stephenson and Elder, 2006)

$$\begin{aligned}
e^{SF}_{xx} &= Q_{11}P_x^2 + Q_{12}(P_y^2+P_z^2), && \text{(S.33)}\\
e^{SF}_{yy} &= Q_{11}P_y^2 + Q_{12}(P_x^2+P_z^2), && \text{(S.34)}\\
e^{SF}_{zz} &= Q_{11}P_z^2 + Q_{12}(P_x^2+P_y^2), && \text{(S.35)}\\
e^{SF}_{yz} &= Q_{44}P_yP_z, && \text{(S.36)}\\
e^{SF}_{xz} &= Q_{44}P_xP_z, && \text{(S.37)}\\
e^{SF}_{xy} &= Q_{44}P_xP_y, && \text{(S.38)}
\end{aligned}$$

where Q_{11}, Q_{12}, and Q_{44} are the electrostrictive coefficients for a cubic material. These quadratic relations have been found to hold even for polarization values beyond the validity of Eq. (S.32) (Li *et al.*, 2014). Here we use the six-index Voigt notation for the components of the strain and stress tensors (Love, 1944).

For a small dynamic applied field in the y direction ΔE_y, the change in the stress-free strain components are given by

$$\begin{aligned}
\Delta e^{SF}_{xx} &= 2Q_{12}\,\varepsilon_0^2\varepsilon^2E_y\,\Delta E_y, && \text{(S.39)}\\
\Delta e^{SF}_{yy} &= 2Q_{11}\,\varepsilon_0^2\varepsilon^2E_y\,\Delta E_y, && \text{(S.40)}\\
\Delta e^{SF}_{zz} &= 2Q_{12}\,\varepsilon_0^2\varepsilon^2E_y\,\Delta E_y, && \text{(S.41)}\\
\Delta e^{SF}_{yz} &= Q_{44}\,\varepsilon_0^2\varepsilon^2E_z\,\Delta E_y, && \text{(S.42)}\\
\Delta e^{SF}_{xz} &= 0, && \text{(S.43)}\\
\Delta e^{SF}_{xy} &= Q_{44}\,\varepsilon_0^2\varepsilon^2E_x\,\Delta E_y. && \text{(S.44)}
\end{aligned}$$

Thus the Fourier components of these quantities are proportional to those of the electric field,

$$\begin{aligned}
\Delta\hat{e}^{SF}_{xx\,mn}(z) &= 2Q_{12}\,\varepsilon_0^2\varepsilon^2\Delta E_y\,\hat{E}_{y\,mn}(z), && \text{(S.45)}\\
\Delta\hat{e}^{SF}_{yy\,mn}(z) &= 2Q_{11}\,\varepsilon_0^2\varepsilon^2\Delta E_y\,\hat{E}_{y\,mn}(z), && \text{(S.46)}\\
\Delta\hat{e}^{SF}_{zz\,mn}(z) &= 2Q_{12}\,\varepsilon_0^2\varepsilon^2\Delta E_y\,\hat{E}_{y\,mn}(z), && \text{(S.47)}\\
\Delta\hat{e}^{SF}_{yz\,mn}(z) &= Q_{44}\,\varepsilon_0^2\varepsilon^2\Delta E_y\,\hat{E}_{z\,mn}(z), && \text{(S.48)}\\
\Delta\hat{e}^{SF}_{xz\,mn}(z) &= 0, && \text{(S.49)}\\
\Delta\hat{e}^{SF}_{xy\,mn}(z) &= Q_{44}\,\varepsilon_0^2\varepsilon^2\Delta E_y\,\hat{E}_{x\,mn}(z). && \text{(S.50)}
\end{aligned}$$

The total strain is the sum of the stress-free strain and the elastic strain $\mathbf{e^T} = \mathbf{e^{SF}} + \mathbf{e^E}$. Likewise, the changes in these quantities due to the applied field are related by $\Delta\mathbf{e^T} = \Delta\mathbf{e^{SF}} + \Delta\mathbf{e^E}$. The components of elastic strain e^E_{ij} are linearly related to the components of stress X_{ij} (Love, 1944). The same relations apply to the changes in these quantities,

$$\begin{aligned}
\Delta X_{xx} &= c_{11}\Delta e^E_{xx} + c_{12}(\Delta e^E_{yy} + \Delta e^E_{zz}), && \text{(S.51)}\\
\Delta X_{yy} &= c_{11}\Delta e^E_{yy} + c_{12}(\Delta e^E_{xx} + \Delta e^E_{zz}), && \text{(S.52)}\\
\Delta X_{zz} &= c_{11}\Delta e^E_{zz} + c_{12}(\Delta e^E_{xx} + \Delta e^E_{yy}), && \text{(S.53)}\\
\Delta X_{yz} &= c_{44}\Delta e^E_{yz}, && \text{(S.54)}\\
\Delta X_{xz} &= c_{44}\Delta e^E_{xz}, && \text{(S.55)}\\
\Delta X_{xy} &= c_{44}\Delta e^E_{xy}, && \text{(S.56)}
\end{aligned}$$

and to their Fourier components,

$$\begin{aligned}
\Delta\hat{X}_{xx\,mn}(z) &= c_{11}\Delta\hat{e}^E_{xx\,mn}(z)\\
&\quad + c_{12}(\Delta\hat{e}^E_{yy\,mn}(z) + \Delta\hat{e}^E_{zz\,mn}(z)), && \text{(S.57)}\\
\Delta\hat{X}_{yy\,mn}(z) &= c_{11}\Delta\hat{e}^E_{yy\,mn}(z)\\
&\quad + c_{12}(\Delta\hat{e}^E_{xx\,mn}(z) + \Delta\hat{e}^E_{zz\,mn}(z)), && \text{(S.58)}\\
\Delta\hat{X}_{zz\,mn}(z) &= c_{11}\Delta\hat{e}^E_{zz\,mn}(z)\\
&\quad + c_{12}(\Delta\hat{e}^E_{xx\,mn}(z) + \Delta\hat{e}^E_{yy\,mn}(z)), && \text{(S.59)}\\
\Delta\hat{X}_{yz\,mn}(z) &= c_{44}\Delta\hat{e}^E_{yz\,mn}(z), && \text{(S.60)}\\
\Delta\hat{X}_{xz\,mn}(z) &= c_{44}\Delta\hat{e}^E_{xz\,mn}(z), && \text{(S.61)}\\
\Delta\hat{X}_{xy\,mn}(z) &= c_{44}\Delta\hat{e}^E_{xy\,mn}(z), && \text{(S.62)}
\end{aligned}$$

We need total strain components that are compatible with a displacement field, and also match the stress boundary conditions at the surface. There are differential relations between the displacement field components and the total strain components (Love, 1944). Since these are linear, the same relations apply between the changes in these quantities due to the applied field,

$$\Delta e_{xx}^{T} = \frac{d\Delta u_x}{dx}, \tag{S.63}$$
$$\Delta e_{yy}^{T} = \frac{d\Delta u_y}{dy}, \tag{S.64}$$
$$\Delta e_{zz}^{T} = \frac{d\Delta u_z}{dz}, \tag{S.65}$$
$$\Delta e_{yz}^{T} = \frac{d\Delta u_y}{dz} + \frac{d\Delta u_z}{dy}, \tag{S.66}$$
$$\Delta e_{xz}^{T} = \frac{d\Delta u_x}{dz} + \frac{d\Delta u_z}{dx}, \tag{S.67}$$
$$\Delta e_{xy}^{T} = \frac{d\Delta u_x}{dy} + \frac{d\Delta u_y}{dx}. \tag{S.68}$$

Boundary conditions arise on the stress because its components in the z direction must be zero at the surface. These also apply to the changes in stress due to the applied field, e.g. $\Delta X_{zz} = \Delta X_{yz} = \Delta X_{xz} = 0$ at $z = 0$. In addition, the displacements, strains, and stresses must tend to zero at large distances from the illuminated area.

We can use an *ansatz* for the displacement field change $\mathbf{\Delta u}$ that can be made to satisfy these conditions. We assume the same type of in-plane Fourier series with z-dependent components as in the solution for ϕ, Eq. (S.22), with

$$\Delta\hat{u}_{xmn}(z) = -A_x \frac{q_{yn}}{q_{xm}} \exp\left[\frac{-q_{xm}^2(|q_{xm}|+|q_{yn}|)\,z}{q_{yn}^2}\right] \hat{\phi^{ss}}_{mn}, \tag{S.69}$$
$$\Delta\hat{u}_{y_{mn}}(z) = -A_y \exp[-(|q_{xm}|+|q_{yn}|)\,z]\,\hat{\phi^{ss}}_{mn}, \tag{S.70}$$
$$\Delta\hat{u}_{zmn}(z) = iA_z \frac{|q_{xm}|+|q_{yn}|}{q_{yn}} \exp\left[\frac{-q_{yn}^2\,z}{|q_{xm}|+|q_{yn}|}\right] \hat{\phi^{ss}}_{mn}. \tag{S.71}$$

where A_x, A_y, and A_z are real constants that will be chosen below to satisfy the boundary conditions. The Fourier components of the changes in total strain can be evaluated using Eqs. (S.63) - (S.68) to be

$$\Delta\hat{e}_{xxmn}^{T}(z) = -iA_x q_{yn} \exp\left[\frac{-q_{xm}^2(|q_{xm}|+|q_{yn}|)\,z}{q_{yn}^2}\right] \hat{\phi^{ss}}_{mn}, \tag{S.72}$$
$$\Delta\hat{e}_{yy_{mn}}^{T}(z) = -iA_y q_{yn} \exp[-(|q_{xm}|+|q_{yn}|)\,z]\,\hat{\phi^{ss}}_{mn}, \tag{S.73}$$
$$\Delta\hat{e}_{zzmn}^{T}(z) = -iA_z q_{yn} \exp\left[\frac{-q_{yn}^2\,z}{|q_{xm}|+|q_{yn}|}\right] \hat{\phi^{ss}}_{mn}, \tag{S.74}$$
$$\Delta\hat{e}_{yz_{mn}}^{T}(z) = \Big(A_y[|q_{xm}|+|q_{yn}|]\exp[-(|q_{xm}|+|q_{yn}|)\,z] - A_z[|q_{xm}|+|q_{yn}|]\exp\left[\frac{-q_{yn}^2\,z}{|q_{xm}|+|q_{yn}|}\right]\Big) \hat{\phi^{ss}}_{mn}, \tag{S.75}$$
$$\Delta\hat{e}_{xzmn}^{T}(z) = \left(A_x \frac{q_{xm}(|q_{xm}|+|q_{yn}|)}{q_{yn}} \exp\left[\frac{-q_{xm}^2(|q_{xm}|+|q_{yn}|)\,z}{q_{yn}^2}\right] - A_z \frac{q_{xm}(|q_{xm}|+|q_{yn}|)}{q_{yn}} \exp\left[\frac{-q_{yn}^2\,z}{|q_{xm}|+|q_{yn}|}\right]\right) \hat{\phi^{ss}}_{mn}, \tag{S.76}$$
$$\Delta\hat{e}_{xy_{mn}}^{T}(z) = -i\left(A_x \frac{q_{yn}^2}{q_{xm}} \exp\left[\frac{-q_{xm}^2(|q_{xm}|+|q_{yn}|)\,z}{q_{yn}^2}\right] + A_y q_{xm} \exp[-(|q_{xm}|+|q_{yn}|)\,z]\right) \hat{\phi^{ss}}_{mn}. \tag{S.77}$$

For the boundary conditions $\Delta X_{zz} = 0$, $\Delta X_{yz} = 0$, and $\Delta X_{xz} = 0$ to be met at all positions (x,y) in the plane of the surface, the $z = 0$ value of their Fourier components must be zero for all (m,n). Substituting the Fourier components $\Delta\hat{e}_{ij_{mn}}^{E}(z) = \Delta\hat{e}_{ij_{mn}}^{T}(z) - \Delta\hat{e}_{ij\ mn}^{SF}(z)$ from Eqs. (S.24)-(S.26), Eqs. (S.45)-(S.50) and Eqs. (S.72)-(S.77) into Eqs. (S.57)-(S.62), the boundary conditions $\Delta\hat{X}_{zzmn}(0) = 0$, $\Delta\hat{X}_{yz_{mn}}(0) = 0$, and $\Delta\hat{X}_{xzmn}(0) = 0$, respectively, are satisfied for all (m,n) when

$$c_{11}A_z + c_{12}(A_x + A_y) = 2\varepsilon_0^2\varepsilon^2[c_{11}Q_{12} + c_{12}(Q_{11}+Q_{12})]\Delta E_y, \tag{S.78}$$
$$A_y - A_z = \varepsilon_0^2\varepsilon^2 Q_{44}\Delta E_y, \tag{S.79}$$
$$A_x - A_z = 0, \tag{S.80}$$

which gives

$$A_x = A_z = 2\varepsilon_0^2\varepsilon^2 \frac{c_{11}Q_{12} + c_{12}(Q_{11}+Q_{12}-Q_{44}/2)}{c_{11}+2c_{12}} \Delta E_y, \tag{S.81}$$
$$A_y = 2\varepsilon_0^2\varepsilon^2 \times \frac{c_{11}(Q_{12}+Q_{44}/2) + c_{12}(Q_{11}+Q_{12}+Q_{44}/2)}{c_{11}+2c_{12}} \Delta E_y. \tag{S.82}$$

The change in the tilt of the surface around the x axis in response to the dynamic applied field is $\Delta\chi = d\Delta u_z/dy$. The

Table S.2 Symmetry of functions.

		Symmetry Type			
		1	2	3	4
$f(x,y)$	x parity	even	odd	even	odd
	y parity	even	even	odd	odd
	type	real	real	real	real
$\hat{f}(q_x,q_y)$	q_x parity	even	odd	even	odd
	q_y parity	even	even	odd	odd
	type	real	imag	imag	real
Static	Scalar	ϕ			
	Vector	E_z<br>u_z	E_x<br>u_x	E_y<br>u_y	
	Tensor	e_{xx}, e_{yy}, e_{zz}<br>X_{xx}, X_{yy}, X_{zz}	e_{xz}<br>X_{xz}	e_{yz}<br>X_{yz}	e_{xy}<br>X_{xy}
Dynamic	Vector	Δu_y<br>$\Delta\chi$		Δu_z	Δu_x<br>$\Delta\eta$
	Tensor	Δe_{yz}<br>ΔX_{yz}	Δe_{xy}<br>ΔX_{xy}	Δe_{xx}, Δe_{yy}, Δe_{zz}<br>ΔX_{xx}, ΔX_{yy}, ΔX_{zz}	Δe_{xz}<br>ΔX_{xz}

Fourier components of this quantity can be evaluated as

$$\hat{\Delta\chi}_{mn}(z) = -A_z[|q_{xm}|+|q_{yn}|]\exp\left[\frac{-q_{yn}^2 z}{|q_{xm}|+|q_{yn}|}\right]\hat{\phi^{ss}}_{mn}. \tag{S.83}$$

The change in the tilt $\Delta\chi$ has maximum magnitude at $x=y=z=0$ which can be evaluated as

$$\Delta\chi_0 = -0.793\left(\frac{1}{\sigma_x}+\frac{1}{\sigma_y}\right)\times 2\varepsilon_0^2\varepsilon^2\left(\frac{c_{11}Q_{12}+c_{12}(Q_{11}+Q_{12}-Q_{44}/2)}{c_{11}+2c_{12}}\right)\Delta E_y\,\phi_{pk}. \tag{S.84}$$

This is linearly proportional to applied dynamic field ΔE_y and the peak static field from the X rays, ϕ_{pk}, and inversely proportional to the beam width σ_x, σ_y.

The predicted surface tilt around the y axis in response to the dynamic applied field is $\Delta\eta = d\Delta u_z/dx$. This is zero at the center $x=y=0$, owing to the assumed symmetry of the static and dynamic electric fields.

Table S.2 shows the symmetries of the various functions calculated. There are four types of symmetries, determined by the parities in the x and y directions. The parities of the reciprocal space functions $\hat{f}(q_x,q_y)$ are the same as those of the real space functions $f(x,y)$. Because the real-space functions are all real, the corresponding reciprocal space functions have either real or imaginary type as shown. Each of the components of vectors or tensors has its own symmetry, as shown. The symmetries of the dynamic function components produced by ΔE_y differ from those of the corresponding static function components. All of the types of strains (total, stress-free, and elastic) have the same symmetries of their components. Only the functions that are even in both x and y, such as $\Delta\chi$, are non-zero at the center of the illuminated area, $x=y=0$. Other functions, such as Δe^T_{zz}, that are zero at the center will have minimal influence on the diffracted beam, so for example we do not predict significant dynamic radial motion of the Bragg peak. There should be significant static radial motion due to e^T_{zz}.

Table S.3 Parameter values used in calculating stress, strain, and displacement distributions due to dynamic field (Ahart *et al.*, 2007; Hoover *et al.*, 1997; Uchino *et al.*, 1980). Q_{44} was calculated from the value of $Q_{33} = 1.15\times10^{-2}$ m^4 C^{-2} for the (1 1 1) direction (Lee *et al.*, 1999), using $Q_{44} = (3Q_{33}-Q_{11}-2Q_{12})/2$ (Li *et al.*, 2014).

$Q_{11} = 2.5\times10^{-2}$ m^4 C^{-2}	$c_{11} = 1.56\times10^{11}$ N m^{-2}
$Q_{12} = -9.6\times10^{-3}$ m^4 C^{-2}	$c_{12} = 0.76\times10^{11}$ N m^{-2}
$Q_{44} = 1.43\times10^{-2}$ m^4 C^{-2}	$c_{44} = 0.68\times10^{11}$ N m^{-2}
$\varepsilon = 2.5\times10^4$	$\varepsilon_0 = 8.85\times10^{-12}$ C V^{-1} m^{-1}
$\phi_{pk} = 10$ V	$\Delta E_y = 1.75\times10^4$ V m^{-1}

E. Results for PMN

We have calculated the distributions of the components of dynamic stress, strain, and displacement and the dynamic tilt, using the parameter values in Tables S.1 and S.3. The distributions are shown in Figs. S.7 - S.11. For these parameters, the net effective electrostrictive coefficient in Eq. (S.84) has a value of

$$\frac{c_{11}Q_{12}+c_{12}(Q_{11}+Q_{12}-Q_{44}/2)}{c_{11}+2c_{12}} = -2.8\times10^{-3}\,\mathrm{m^4C^{-2}}, \tag{S.85}$$

giving a peak-peak tilt of $\chi_{pp} = +5.2\times10^{-5}$ for a peak-peak field of $E_{pp} = 3.5\times10^4$ V/m, which is the maximum peak-peak amplitude of the applied field under our experimental conditions. Because we use an inward-pointing z axis, positive ΔE_y corresponds to a tilt of the surface normal towards $+y$, which is the direction of negative applied potential.

Because the stress-free strain distributions follow those of the electric field, they are confined to the region near the illuminated surface. However, some of the components of total strain, stress, and displacement extend further in x, y, or z. The region of significant tilt $\Delta\chi$ is confined to the region near the illuminated surface. The effective penetration depth of the 7.4 keV X rays is $\ell_{abs}\sin\theta_B/2 = 1.2\times10^{-6}$ m, accounting for absorption of both the incident and exit beam. Since this is smaller than the beam size, the illuminated volume has an average tilt similar to the maximum value. The average of the χ_{pp} distribution weighted by the effective X-ray intensity $I_0(x,y)\exp(-2z/\ell_{abs}\sin\theta_B)$ is 1.2×10^{-5} at $E_{pp} = 3.5\times10^4$ V m^{-1}. Note that the predicted motions of the sample surface in the in-plane directions Δu_x and Δu_y are much smaller than the illuminated area, and so would not contribute to the speckle decorrelation.

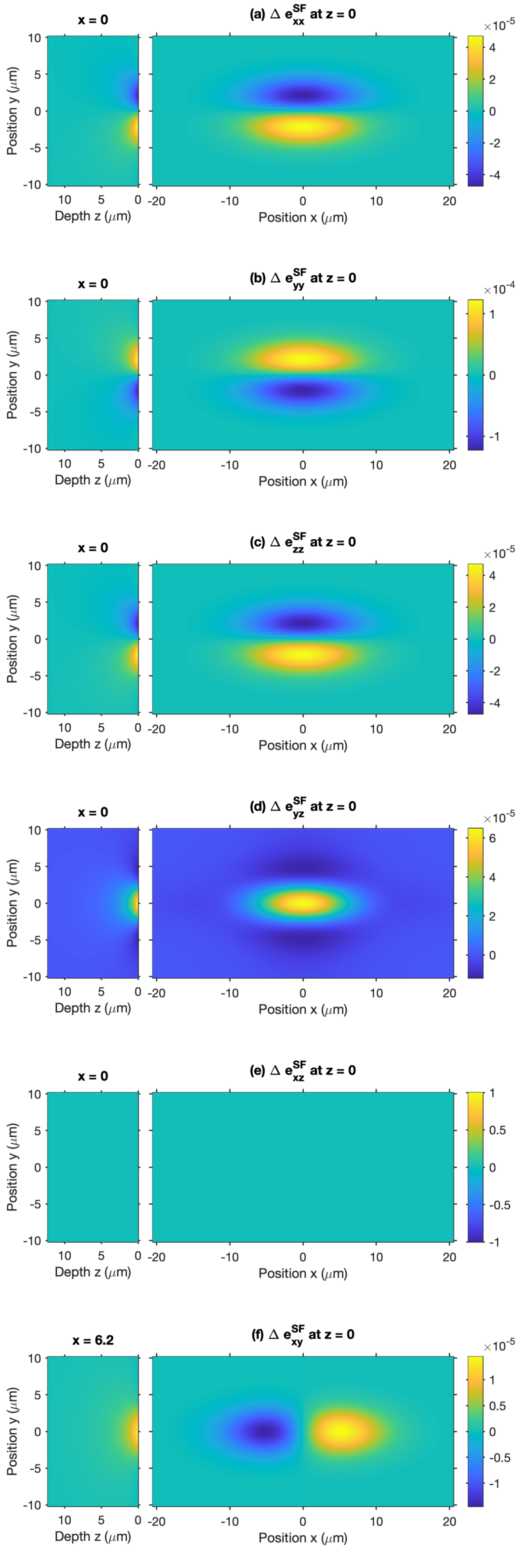

Figure S.7 Components of the dynamic stress-free strain tensor Δe^{SF} at the surface (right) and inside sample (left) at $\Delta E_y = 1.75 \times 10^4$ V m^{-1} and $\phi_{pk} = 10$ V.

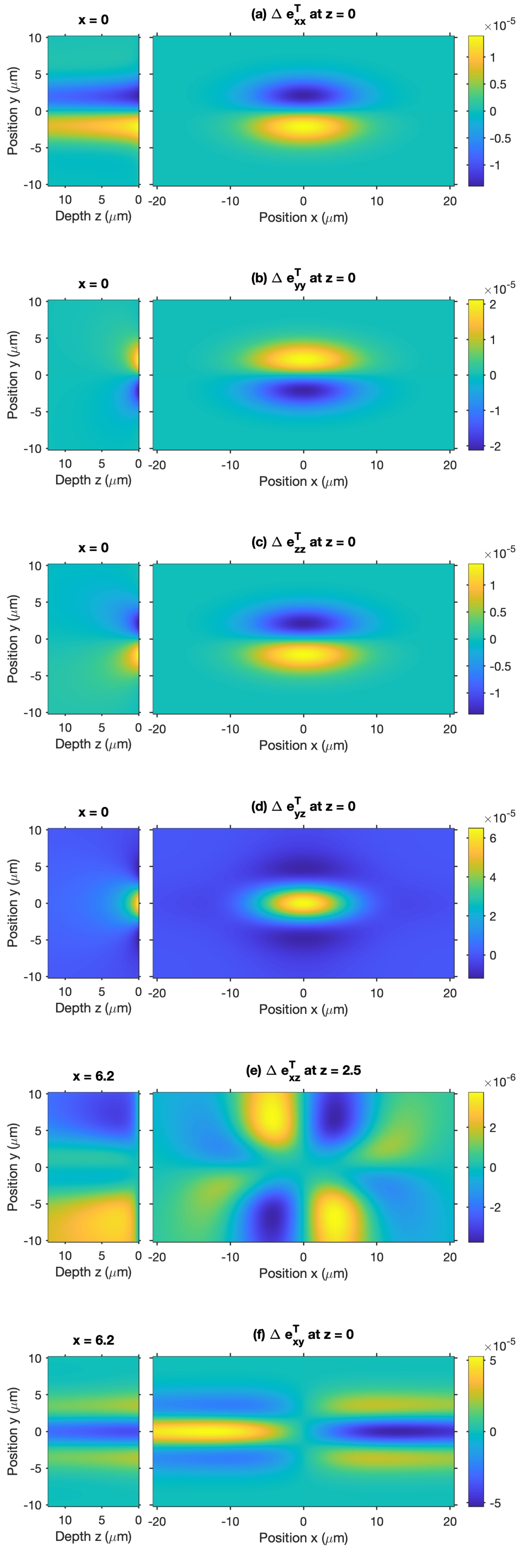

Figure S.8 Components of the dynamic total strain tensor Δe^{T} near the surface (right) and inside sample (left) at $\Delta E_y = 1.75 \times 10^4$ V m^{-1} and $\phi_{pk} = 10$ V.

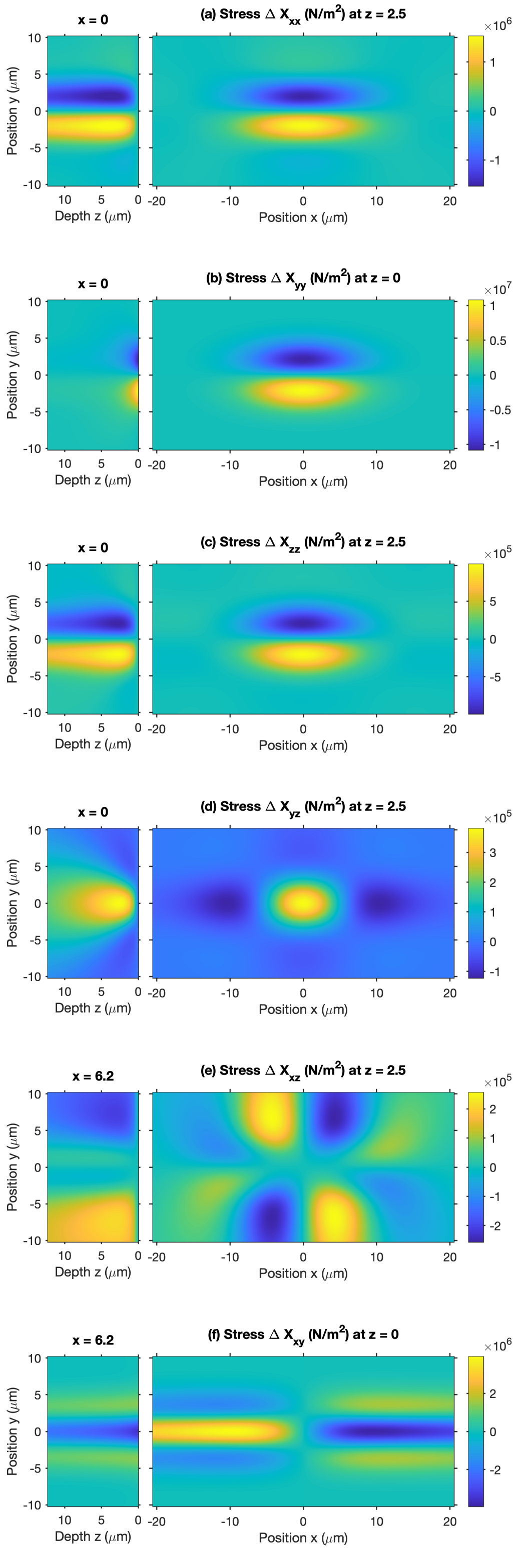

Figure S.9 Components of the dynamic stress tensor ΔX near the surface (right) and inside sample (left) at $\Delta E_y = 1.75 \times 10^4$ V m^{-1} and $\phi_{pk} = 10$ V.

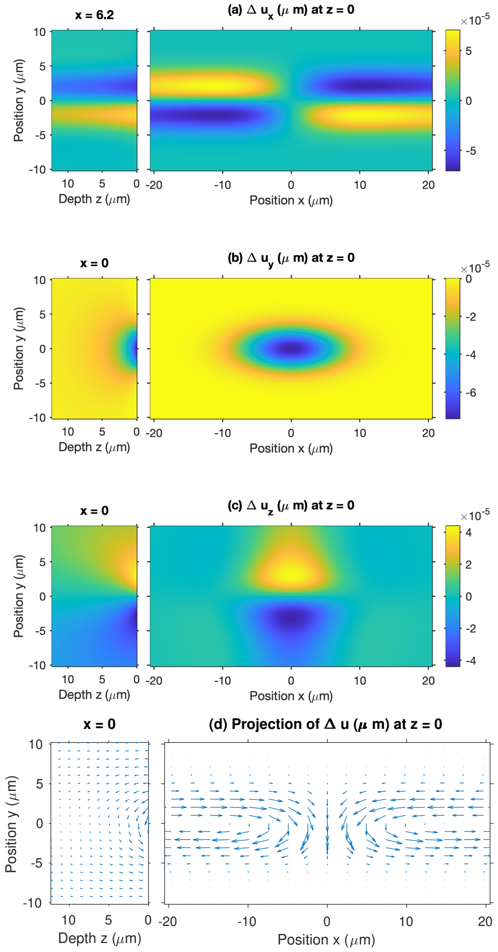

Figure S.10 Components of the dynamic displacement vector Δu at the surface (right) and inside sample (left) at $\Delta E_y = 1.75 \times 10^4$ V m^{-1} and $\phi_{pk} = 10$ V.

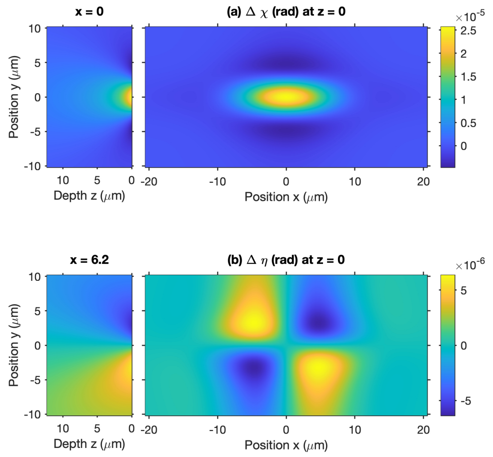

Figure S.11 Dynamic tilts $\Delta\chi$ and $\Delta\eta$ at the surface (right) and inside sample (left) at $\Delta E_y = 1.75 \times 10^4$ V m^{-1} and $\phi_{pk} = 10$ V.

ACKNOWLEDGMENTS

The XPCS experiment, data analysis and interpretation were supported by the U.S. Department of Energy, Office of Science, Basic Energy Sciences, Materials Science and Engineering Division. The experiments were performed mostly at beamline 8-ID-E, and also beamlines 12-ID-D and 33-BM of the Advanced Photon Source, a U.S. Department of Energy (DOE) Office of Science User Facility operated for the DOE Office of Science by Argonne National Laboratory under Contract No. DE-AC02-06CH11357. Work performed at the Center for Nanoscale Materials, a U.S. Department of Energy Office of Science User Facility, was supported by the U.S. DOE, Office of Basic Energy Sciences, under Contract No. DE-AC02-06CH11357. BXW and ZGY were supported by the Natural Sciences and Engineering Research Council of Canada (NSERC, Discovery Grant No. RGPIN-2023-04416).

We thank Hua Zhou at the Advanced Photon Source for helpful discussions and instrumentation expertise.

REFERENCES

Ahart, Muhtar, Aravind Asthagiri, Zuo-Guang Ye, Przemyslaw Dera, Ho-kwang Mao, Ronald E. Cohen, and Russell J. Hemley (2007), "Brillouin scattering and molecular dynamics study of the elastic properties of $Pb(Mg_{1/3}Nb_{2/3})O_3$," Physical Review B **75** (14), 144410.

Bras, Wim, Dean D. A. Myles, and Roberto Felici (2021), "When x-rays alter the course of your experiments," J. Phys. Condens. Matter **33**, 423002.

Griffiths, David J (1999), *Introduction to Electrodynamics*, 3rd ed. (Pearson).

Gullickson, Eric (2010), "X-ray interactions with matter," `https://henke.lbl.gov/optical_constants` last accessed on February 20, 2022.

Hoover, B D, B. A. Tuttle, W. R. Olson, G. M. Goy, R. A. Brooks, and C. F. King (1997), *Evaluation of Field Enforced Antiferroelectric to Ferroelectric Phase Transition Dielectrics and Relaxor Ferroelectrics for Pulse Discharge Capacitors*, Tech. Rep. SAND97-2295 (Sandia National Laboratory).

Hruszkewycz, S O, M. Sutton, P. H. Fuoss, B. Adams, S. Rosenkranz, K. F. Ludwig, W. Roseker, D. Fritz, M. Cammarata, D. Zhu, S. Lee, H. Lemke, C. Gutt, A. Robert, G. Grübel, and G. B. Stephenson (2012), "High contrast x-ray speckle from atomic-scale order in liquids and glasses," Phys. Rev. Lett. **109**, 185502.

Lee, Sang-Goo, Ralph G. Monteiro, Robert S. Feigelson, Howard S. Lee, Myeongkyu Lee, and Seung-Eek Park (1999), "Growth and electrostrictive properties of $Pb(Mg_{1/3}Nb_{2/3})O_3$ crystals," Applied Physics Letters **74** (7), 1030–1032.

Li, Fei, Li Jin, Zhuo Xu, and Shujun Zhang (2014), "Electrostrictive effect in ferroelectrics: An alternative approach to improve piezoelectricity," Applied Physics Reviews **1** (1), 011103.

Love, A E H (1944), *A Treatise on the the Mathematical Theory of Elasticity*, 4th ed. (Dover Publications) 1st American Printing of 1927 4th edition (by Special Arrangement with the Cambridge University Press and The Macmillan Co.).

Moulder, John, F, William F. Stickle, Peter E. Sobol, and Kenneth D. Bomben (1992), *Handbook of X-ray Photoelectron Spectroscopy* (Perkin-Elmer Corporation).

Stephenson, G B, and K. R. Elder (2006), "Theory for equilibrium 180° stripe domains in $PbTiO_3$ films," Journal of Applied Physics **100** (5), 051601.

Tielsch, Brian J, and Julia E. Fulghum (1996), "Differential charging in XPS. part I: Demonstration of lateral charging in a bulk insulator using imaging XPS," Surface and Interface Analysis **24**, 422–427.

Uchino, K, S. Nomura, L. E. Cross, S. J. Jang, and R. E. Newnham (1980), "Electrostrictive effect in lead magnesium niobate single crystals," Journal of Applied Physics **51** (2), 1142–1145.

Yasuno, Satoshi (2019), "Charge compensation in hard x-ray photoelectron spectroscopy by electron beam of several kilo-electron-volts," Journal of Surface Analysis **26**, 202–203.
